# Synthesis and Characterization of New N-acyl Hydrazone Derivatives of Carprofen as Potential Tuberculostatic Agents

**DOI:** 10.3390/antibiotics13030212

**Published:** 2024-02-23

**Authors:** Ilinca Margareta Vlad, Diana Camelia Nuță, Miron Theodor Căproiu, Florea Dumitrașcu, Eleonóra Kapronczai, Georgiana Ramona Mük, Speranta Avram, Adelina Gabriela Niculescu, Irina Zarafu, Vanesa Alexandra Ciorobescu, Ana Maria Brezeanu, Carmen Limban

**Affiliations:** 1 Department of Pharmaceutical Chemistry, Faculty of Pharmacy, “Carol Davila” University of Medicine and Pharmacy, Traian Vuia no. 6, 020956 Bucharest, Romania; ilinca.vlad@umfcd.ro (I.M.V.); vanesa-alexandra.buzatu@rez.umfcd.ro (V.A.C.); ana.brezeanu@stud.umfcd.ro (A.M.B.); carmen.limban@umfcd.ro (C.L.); 2 “C. D. Nenitzescu” Institute of Organic and Supramolecular Chemistry, 202B Splaiul Independenței, 060023 Bucharest, Romania; dorucaproiu@gmail.com (M.T.C.); fdumitra@yahoo.com (F.D.); 3 Department of Chemistry, Supramolecular Organic and Organometallic Chemistry Centre, Faculty of Chemistry and Chemical Engineering, Babeş-Bolyai University, 11 Arany János, 400028 Cluj-Napoca, Romania; erzsebet.denes@ubbcluj.ro; 4 Faculty of Biology, University of Bucharest, Splaiul Independenței 91-95, 050095 Bucharest, Romania; mukramonageorgiana@yahoo.com (G.R.M.); speranta.avram@bio.unibuc.ro (S.A.); 5 ”St. Stephen’s” Pneumoftiziology Hospital, Șos. Ștefan cel Mare 11, 020122 Bucharest, Romania; 6 Research Institute of the University of Bucharest, Sos. Panduri 90-92, 050095 Bucharest, Romania; adelina.niculescu@upb.ro; 7 Department of Science and Engineering of Oxide Materials and Nanomaterials, National University of Science and Technology Politehnica Bucharest, 011061 Bucharest, Romania; 8 Department of Organic Chemistry, Biochemistry and Catalysis, Faculty of Chemistry, University of Bucharest, 4-12 Regina Elisabeta, 030018 Bucharest, Romania; zarafuirina@yahoo.fr

**Keywords:** N-acyl hydrazone derivatives, microwave synthesis, FT-IR spectral data, NMR analysis, HRMS analysis, tuberculostatic activity

## Abstract

N-acyl hydrazone (NAH) is recognized as a promising framework in drug design due to its versatility, straightforward synthesis, and attractive range of biological activities, including antimicrobial, antitumoral, analgesic, and anti-inflammatory properties. In the global context of increasing resistance of pathogenic bacteria to antibiotics, NAHs represent potential solutions for developing improved treatment alternatives. Therefore, this research introduces six novel derivatives of (*EZ*)-N’-benzylidene-2-(6-chloro-9*H*-carbazol-2-yl)propanehydrazide, synthesized using a microwave-assisted method. In more detail, we joined two pharmacophore fragments in a single molecule, represented by an NSAID-type carprofen structure and a hydrazone-type structure, obtaining a new series of NSAID-N-acyl hydrazone derivatives that were further characterized spectrally using FT-IR, NMR, and HRMS investigations. Additionally, the substances were assessed for their tuberculostatic activity by examining their impact on four strains of *M. tuberculosis*, including two susceptible to rifampicin (RIF) and isoniazid (INH), one susceptible to RIF and resistant to INH, and one resistant to both RIF and INH. The results of our research highlight the potential of the prepared compounds in fighting against antibiotic-resistant *M. tuberculosis* strains.

## 1. Introduction

The principal pharmacophore scaffold, N-acyl hydrazone (NAH), consists of both amide and imine functional groups, potentially displaying geometrical and conformational stereoisomerism [1]. Rotation along the imine bond can generate two geometrical stereoisomers, the *E* and *Z* forms, while rotation across the amide bond can lead to conformational stereoisomerism, NAH being able to exist as *syn*periplanar and *anti*periplanar conformers [2].

NAHs have demonstrated remarkable versatility, showing promising outcomes in both drug design and drug chemistry. This adaptability is attributed to the straightforward synthesis of NAHs. Typically, these compounds are created by combining aldehydes or ketones with hydrazides. Notably, their distinctive structural characteristics encompass the presence of two geometric isomers, *E* and *Z*, in relation to the C=N bond plane. Additionally, there are two conformers arising from the rotation around the C-N bond within the amidic group.

The N-acyl hydrazone fragment has been identified in a large number of compounds acting on different targets [3,4,5,6,7,8]. NAHs are recognized for their antibacterial [9,10,11,12], antimycobacterial [13,14,15], antiprotozoal [16,17,18,19,20], antiviral [21], antitumoral [22], analgesic, and anti-inflammatory properties [23].

**Figure 1:**
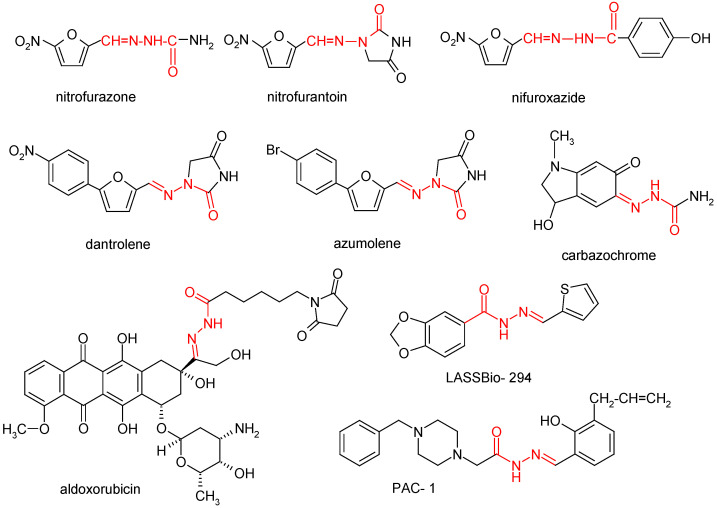
N-acyl hydrazone derivatives with biological applications.

There exist medicinal substances based on NAH (Figure 1), such as nitrofurazone (broad-spectrum topical antibacterial agent) [24]; nitrofurantoin (oral antibacterial agent for treating gastrointestinal tract infections) [25]; nifuroxazide (antibacterial agent of use with gastrointestinal infections); carbazochrome (hemostatic agent, tested with good results against hereditary hemorrhagic telangiectasia) [26,27]; dantrolene and azumolene (approved for the treatment of malign hyperthermia) [28,29]; aldoxorubicin (a hydrazone derivative of doxorubicin, specifically a maleinimidoalkanoyl hydrazone), which functions as an albumin-binding prodrug of doxorubicin and reached phase III clinical trials for treating metastatic soft tissue sarcoma with deep localization—it releases doxorubicin at the target site with fewer systemic adverse effects, including lack of cardiotoxicity [30]; LASSBio-294 ((2-thienylidene)-3,4-methylenedioxybenzoylhydrazine) (under preclinical testing for the treatment of heart failure) [31,32]; and PAC-1 ((4-benzylpiperazino)acetic acid-(3-allyl-2-hydroxybenzylidene)hydrazide) (procaspase activator that reached clinical studies testing stage in 2015). In terms of the correlation between structure and activity, the segment of PAC-1 denoted *orto*-hydroxy-N-acyl hydrazone plays a crucial role in binding zinc and triggering the activation of procaspase 3 [33,34].

When examining compounds containing the NAH fragment, it is essential to assess their stability. The stability of these compounds, featuring diverse substituents on the amide nitrogen and the imine carbon, has been investigated. The physicochemical profiles of N-methyl-N-acyl hydrazones exhibit superior stability and water solubility, attributed to conformational changes. Another critical factor is the stability of the imine double bond, typically adopting an *E*-type spatial configuration in N-acyl hydrazones, though a *Z*-type configuration is also feasible. In vivo stability studies indicate that interconversion of the C=N bond configuration is not feasible [35].

Numerous investigations have demonstrated the enhanced efficacy of drugs incorporating the NAH fragment compared to the original drug. For example, Effenberger et al. [36] reported that a doxorubicin hybrid, incorporating an N-acyl hydrazone moiety, displayed superior anticancer effects in comparison to unmodified doxorubicin, showcasing a distinct mode of action.

Presently, the resistance of pathogenic bacteria to antibiotics employed in therapy is acknowledged as a significant issue posing a threat to public health. Consequently, there is a pressing requirement to formulate novel compounds with varied mechanisms of action to address the escalating danger posed by multidrug-resistant bacteria.

*Mycobacterium tuberculosis* infection is still prevalent at the global level, infecting one third of the world’s population and representing one of the top causes of infection-related mortality, with ~29% of deaths being correlated to its resistance to currently available drugs [37,38,39,40,41]. Many factors, such as late diagnosis; inappropriate treatment selection, drug supply, and/or drug concentration; and poor patient compliance, have played a role in the rise of strains exhibiting multiple drug resistance (MDR), extensive drug resistance (XDR), extreme drug resistance (XXDR), or, in some cases, total drug resistance (TDR) [42,43,44,45]. This threatening context requires the urgent development of novel therapeutic strategies to reduce the mortality and morbidity burden and move one step closer to the aim of the World Health Organization (WHO) to achieve a 95% decrease in mortality due to *M. tuberculosis* infection and a 90% decrease in new cases by 2035 [46]. Several new antibiotics (bedaquiline, delamanid, and pretomanid) have received approval for the treatment of *M. tuberculosis* MDR infections [37,47], but unfortunately, resistance has already merged with these new compounds [48,49]. Derivatives of existing anti-tuberculosis medications, such as ethambutol, and repurposed drugs, such as linezolid and clofazimine, have also been proposed but have been threatened by the quick emergence of resistance [43,49,50,51].

**Figure 2:**
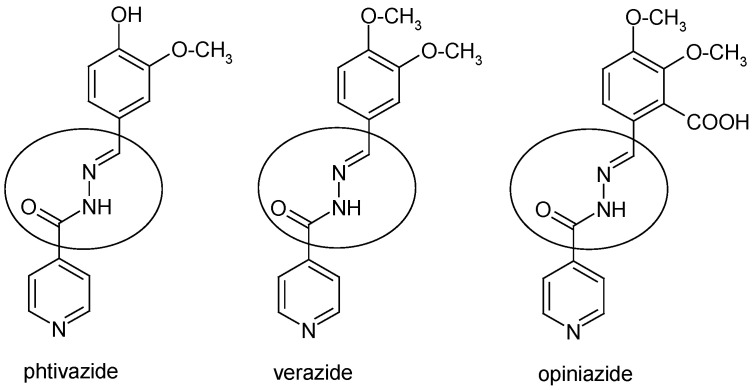
Chemical structure of anti-tuberculosis drugs with NAH pharmacophore moiety.

Figure 2 presents the medicinal substances with tuberculostatic activity that contain NAH scaffolds.

Six N-acyl hydrazones incorporating vitamin B6 have been successfully synthesized using pyridoxal hydrochloride and N-acyl hydrazine. The characterization of all synthesized compounds has been completed, and their efficacy against *M. tuberculosis* has been evaluated. Notably, the N-acyl hydrazone with *para*-pyridine substitution exhibited the most potent activity [52].

Certain N’-benzylidene-2-oxo-2*H*-chromene-3-carbohydrazides demonstrated noteworthy activity in comparison to first-line drugs like pyrazinamide [14].

New acyl hydrazones based on 2*H*-chromene or coumarin were synthesized and assessed for their in vitro antimycobacterial efficacy against the reference strain *M. tuberculosis* H37Rv. Considering the results obtained, these compounds exhibit potential as hybrid anti-tuberculosis agents [53].

Furoxanyl N-acyl hydrazone derivatives were identified as promising lead compounds for tuberculosis treatment, even against resistant strains. These derivatives displayed activity against a clinical isolate of a multidrug-resistant tuberculosis (MDR-TB) strain [54].

From a range of isonicotinoyl hydrazone derivatives, a potential lead compound can be identified for the design and development of more effective antimycobacterial agents. Antimycobacterial results indicated that compounds (*E*)-N’-(2-ethoxybenzylidene) isonicotinohydrazide and (*E*)-N’-(2-fluorobenzyliden)isonicotinohydrazide demonstrated higher effectiveness against *M. tuberculosis* H37Rv and two clinical isolates when compared to isoniazid [55].

Novel derivatives of 2-thiophenecarboxylic acid hydrazide have been obtained, and among them, 2-thiophenecarbonylhydrazone-5,7-dibromoisatin exhibited the most substantial activity against *M. tuberculosis* H37Rv [56].

On the other hand, carprofen, a nonsteroidal anti-inflammatory drug for veterinary use, has been identified as having the capacity to impede efflux pump activity in *M. tuberculosis*, influence the mycobacterial biofilm phenotype, and interfere with the membrane potential of mycobacteria. Consequently, carprofen and a chemical analogue, 2-(6-chloro-9*H*-carbazol-3-yl) acetic acid, possess the potential to alter tuberculostatic drug resistance through their diverse mechanisms of action. These findings propose alternative pathways compared to existing first-line anti-TB drugs, facilitating a rapid progression to clinical trials for novel drug combinations [57].

Furthermore, the inhibition of neutrophil phagocytosis, a characteristic associated with the anti-inflammatory activity of carprofen [58], could hold significant implications in the clinical treatment of tuberculosis. This is particularly noteworthy as the primary pathway of *M. tuberculosis* pathogenesis relies on the initial phagocytic uptake of bacteria by host cells.

In 2017, a study was conducted exploring the coumarin-carprofen skeleton [59]. The compounds had excellent in vitro activity on *M. tuberculosis* H37Rv, with an MIC of 1.56 μg/mL (13 times more active than pyrazinamide or ciprofloxacin, with MICs of 3.125 μg/mL).

Continuing our research [60,61,62], through molecular hybridization, we joined two pharmacophore fragments in a single molecule, represented by an NSAID-type carprofen structure and a hydrazone-type structure, obtaining a new series of NSAID-N-acyl hydrazone derivatives (**1a**–**f**).

## 2. Results

**Scheme 1:**
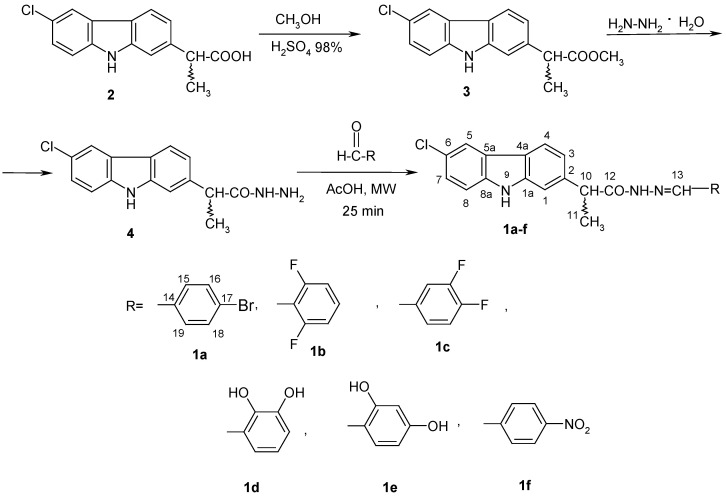
Synthetic pathway for the novel derivatives of (*EZ*)-N’-benzylidene-2-(6-chloro-9*H*-carbazol-2-yl)propanehydrazide.

Six new derivatives of (*EZ*)-N’-benzylidene-(2*RS*)-2-(6-chloro-9*H*-carbazol-2-yl)propanehydrazide were synthesized according to Scheme 1.

### 2.1. Spectral Data

**(*EZ*)-N’-(4-bromobenzylidene)-2-(6-chloro-9*H*-carbazol-2-yl)propanehydrazide (1a)**; m.p. 215–222 °C; yield 63%.

**^1^H-NMR** (500 MHz, DMSO-d6, δ ppm, J Hz): 11.66, 11.39 (s, H-9); 11.37, 11.34 (s, HN); 8.17, 7.88 (s, H-13); 8.16, 8.13 (d, H-5, 1.9); 8.09, 8.05 (d, H-4, 1.8, 8.2); 7.63 ÷ 7.61 (m, 4H, H-15, H-19, H-16, H-18); 7.49 (bs, 1H, H-1); 7.46 (d, 1H, H-8, 8.6); 7.34 (m, 1H, H-7); 7.19 (bd, H-3, 8.2); 4.81, 3.86 (q, H-10, 7.0); 1.50, 1.47 (d, H-11, 7.0) (Figure S1).

**^13^C-NMR** (125 MHz, DMSO-d6, δ ppm): 175.14, 172.89, 169.96 (C-12); 145.36, 141.30 (C-13); 140.54 (C-8a); 140.44 (C-1a); 139.99 (C-14); 138.31, 138.25 (C-2); 133.62, 133.57 (C-17); 131.78, 131.74 (C-16, C-18); 128.79, 128.55 (C-15, C-19); 125.05, 124.97 (C-7); 123.59 (C-5a); 122.82, 122.78 (C-4a); 120.41, 120.31 (C-6); 119.55, 119.48 (C-5); 120.18, 119.65, (C-4); 119.02, 118.75 (C-3); 112.31, 112.26 (C-8); 109.85, 109.62 (C-1); 44.43, 43.61, 41.16 (C-10); 18.84, 18.68 (C-11) (Figure S2).

**FT-IR** (ATR in solid, ν cm^−1^): 3409m; 3241m; 3046w; 2978w; 1659vs; 1621sh; 1598w; 1535m; 1470m; 1346m; 1273w; 1229m; 1186m; 1063m; 1006w; 948w; 867w; 816m; 727w.

Chemical formula C_22_H_17_BrClN_3_O, exact mass 453.02435, **HRMS** (APCI+, DMSO+MeOH), *m*/*z* (%): 456.02927 (100) [M+H]^+^, 228.05769 (79) [C_14_H_11_ClN]^+^ (Figures S3 and S4).

**(*EZ*)-N’-(2,6-difluorobenzylidene)-2-(6-chloro-9*H*-carbazol-2-yl)propanehydrazide (1b)**; m.p. 214–221 °C; yield 68%.

For compound **1b,** the *E*/*Z* ratio is 1.0:0.67.

**^1^H-NMR** (500 MHz, DMSO-d6, δ ppm, *J* Hz): 11.70, 11.39 (s, H-9); 11.44, 11.34 (s, HN); 8.36 (s, H-13); 8.17, 8.13 (d, H-5, 1.9); 8.09, 8.05 (d, H-4, 8.2); 7.49 (tt, 1H, H-17, *J*(H^17^-F^15,19^) = 6.6 Hz, *J*(H^17^–H^16,18^) = 9.1 Hz); 7.46 (d, 1H, H-8, 8.6); 7.44 (bs, 1H, H-1); 7.36, 7.34 (dd, 1H, H-7, 1.9, 8.6); 7.22 ÷ 7.14 (m, 2H, H-3, H-16, H-18); 4.79, 3.84 (q, H-10, 7.0); 1.49, 1.47 (d, H-11, 7.0).

H-1, H-8, and H-17 protons can appear together and are difficult to identify, presenting as a multiplet in the area 7.52 ÷ 7.43 (m, 3H, H-1, H-8, H-17).

The isomer with H-10 at δ = 4.79 ppm represents the majority compared to the isomer with δ = 3.84 ppm; ratio 1/0.67 (Figure S5).

**^13^C-NMR** (125 MHz, DMSO-d6, δ ppm): 175.27, 169.89 (C-12); 161.28, 161.23 (dd, C-15 or C-19, ^4^*J*(F^15^-F^19^) = 6 Hz, *J*(C-F) = 254.3 Hz); 140.57, 140.52 (C-8a); 139.98, 139.85 (C-1a); 138.34, 138.29 (C-2); 136.9, 132.92 (d, C-13, *J*(2F^15,19^-C^13^) = 14.6 Hz); 131.63, 131.29 (t, C-17, ^3^*J*(2F-C^17^) = 11.1 Hz); 125.07, 124.98 (C-7); 123.59 (C-5a); 122.84, 122.75 (C-4a); 120.47, 120.47 (C-6); 120.29, 120.22 (C-5); 119.61, 119.49 (C-3); 112.40, 112.26 (dd, C-16 and C-18, ^4^*J*(F^19^-C^16^) = 4.2 Hz, *^vic^J*(F^19^-C^16^) = 15.4 Hz); 120.47 (C-4); 112.26 (C-8); 111.44 (t, C-14, *^vic^J*(2F-C^14^) = 14.0 Hz); 109.87, 109.63 (C-1); 44.64, 40.58 (C-10); 18.86, 18.66 (C-11) (Figure S6).

Simulation results showed that *^vic^J*(F^15^-C^16^) = 21.2 Hz; ^4^*J*(F^19^-C^16^) = 4.2 Hz.

**FT-IR** (ATR in solid, ν cm^−1^): 3327s; 3242sh; 3037m; 2978w; 2926w; 1633vs; 1617sh; 1592vs; 1464vs; 1385m; 1348m; 1240s; 1207sh; 1066m; 1028w; 1000s; 956m; 863w; 791m.

Chemical formula C_22_H_16_ClF_2_N_3_O, exact mass 411.09500, **HRMS** (APCI+, DMSO+MeOH), *m*/*z* (%): 412.10269 (100) [M+H]^+^, 228.05814 (12) [C_14_H_11_ClN]^+^, 191.14304 (7), 158.02701 (6), 141.00042 (10), 77.03822 (5) (Figures S7 and S8).

**(*EZ*)-N’-(3,4-difluorobenzylidene)-2-(6-chloro-9*H*-carbazol-2-yl)propanehydrazide (1c)**; m.p. 224–230 °C; yield 65%.

For compound **1c**, the ratio between the E and Z stereoisomers is 1.0:0.9.

**^1^H-NMR** (500 MHz, DMSO-d6, δ ppm, *J* Hz): 11.67, 11.33 (s, H-9); 11.38 (s, HN); 8.18, 7.87 (s, H-13); 8.16, 8.13 (d, H-5, 1.8); 8.09, 8.06 (d, H-4, 8.2); 7.74, 7.68 (m, 1H, H-15); 7.51-7.44 (m, 4H, H-1, H-8, H-18, H-19); 7.36, 7.32 (dd, H-7, 1.8, 7.6); 7.20, 7.18 (d, H-3, 8.2); 4.83, 3.86 (q, H-10, 7.0); 1.49, 1.47 (d, H-11, 7.0) (Figure S9).

**^13^C-NMR** (125 MHz, DMSO-d6, δ ppm): 175.26, 170.03 (C-12); 150.61 (dd, C-16, *J*(C^16^-F^17^) = 13.8 Hz, *J*(C^16^-F^16^) = 248.8 Hz); 149.33 (dd, C-17, *J*(C^16^-F^17^) = 15.0 Hz, *J*(C^17^-F^17^) = 245 Hz); 144.40, 140.23 (C-13); 140.55 (C-8a); 139.95 (C-1a); 138.32, 138.25 (C-2); 132.18, 132.12 (C-14, *J*(C^14^-F^16^) = 5.7 Hz); 125.05, 124.96 (C-7); 124.10, 124.01 (C-19); 123.59 (C-5a); 122.83, 122.77 (C-4a); 120.18, 119.68 (C-6); 120.53 (C-4); 119.60, 119.55 (C-5); 119.04, 118.74 (C-3); 118.03, 117.91 (d, C-18, *J*(C^18^-F^17^) = 14.8 Hz); 115.32, 114.80 (d, C-15, *J*(C^15^-F^16^) = 19.5 Hz); 112.32, 112.25 (C-8); 109.72, 109.62 (C-1); 44.44, 41.12 (C-10); 18.96, 18.88 (C-11) (Figure S10).

**FT-IR** (ATR in solid, ν cm^−1^): 3348m; 3190w; 3028m; 2912w; 1644vs; 1577s; 1514s; 1466m; 1444m; 1349m; 1279s; 1242m; 1204s; 1065m; 946w; 866w; 811m; 727w; 624m; 594m.

Chemical formula C_22_H_16_ClF_2_N_3_O, exact mass 411.09500, **HRMS** (APCI+, DMSO+MeOH), *m*/*z* (%): 412.10001 (100) [M+H]^+^, 328.11961 (18) [C_18_H_19_ClN_3_O]^+^, 228.05638 (13) [C_14_H_11_ClN]^+^ (Figures S7, S8, S11 and S12).

**(*EZ*)-N’-(2,3-dihydroxybenzylidene)-2-(6-chloro-9*H*-carbazol-2-yl)propanehydrazide (1d)**; m.p. 143–149 °C; yield 70%.

In compound **1d**, stereoisomers E and Z are in a ratio of 1.0:0.35.

**^1^H-NMR** (300 MHz, DMSO-d6, δ ppm, J Hz): 11.79, 11.27 (s, H-9); 11.38, 11.34 (s, HN); 9.19 (bs, OH); 8.35, 8.21 (s, H-13); 8.17, 8.14 (d, H-5, 1.9); 8.10, 8.06 (d, H-4, 8.2); 7.49–7.44 (m, 2H, H-1, H-8); 7.34, 7.33 (dd, H-7, 1.9, 7.6); 7.19, 7.17 (d, H-3, 8.2); 6.89 (dd, 1H, H-19); 6.81, 6.80 (dd, H-17, 1.7, 7.8); 6.70 (t, 1H, H-18, 7.8); 4.72, 3.86 (q, H-10, 7.0); 1.50, 1.46 (d, H-11, 7.0) (Figure S13).

**^13^C-NMR** (75 MHz, DMSO-d6, δ ppm): 174.64, 169.68 (C-12); 147.87, 141.41 (C-13); 145.93, 145.58 (C-14); 145.54, 145.13 (C-15); 140.60, 140.42 (C-8a); 139.88 (C-1a); 138.38, 138.31 (C-2); 125.13, 125.02 (C-7); 123.62 (C-5a); 122.89, 122.82 (C-4a); 120.69, 120.62 (C-16); 120.51, 120.26 (C-6); 119.92, 119.61 (C-5); 119.75, 119.31 (C-4); 119.10, 119.04 (C-19); 118.99, 118.73 (C-3); 118.80 (C-18); 117.29, 117.07 (C-17); 112.38, 112.30 (C-8); 109.69 (C-1); 44.37, 41.28 (C-10); 19.14, 18.90 (C-1) (Figure S14).

**FT-IR** (ATR in solid, ν cm^−1^): 3370m; 3185w; 3043w; 2972w; 2933w; 1642vs; 1555m; 1468s; 1359m; 1266vs; 1198s; 1118m; 1067m; 1007w; 945m; 869w; 761w; 729m; 611m.

Chemical formula C_22_H_18_ClN_3_O_3_, exact mass 407.10367, **HRMS** (APCI+, DMSO+MeOH), *m*/*z* (%): 408.11007 (100) [M+H]^+^, 328.12057 (5) [C_18_H_19_ClN_3_O]^+^ (Figures S15 and S16).

**(*EZ*)-N’-(2,4-dihydroxybenzylidene)-2-(6-chloro-9*H*-carbazol-2-yl)propanehydrazide (1e)**, m.p. 170–175 °C; yield 63%.

For compound **1e**, the ratio between the geometric stereoisomers E and Z is 1.0:3.0.

**^1^H-NMR** (300 MHz, DMSO-d6, δ ppm, *J* Hz): 11.35 (s, H-9); 11.37, 11.29 (s, HN); 11.09 (bs, OH); 10.01 (bs, OH); 9.92 (bs, OH); 9.80 (bs, OH); 8.26, 8.08 (s, H-13); 8.16, 8.13 (d, H-5, 1.9); 8.09, 8.06 (d, H-4, 8.2); 7.49–7.45 (m, 2H, H-1, H-8); 7.45, 7.24 (d, 1H, H-19, 8.4); 7.35, 7.33 (dd, H-7, 1.9, 7.6); 7.19, 7.16 (d, H-3, 8.2); 6.32 (dd, 1H, H-18, 1.9, 8.4); 6.28 (d, 1H, H-16, 1.9); 4.65, 3.83 (q, H-10, 7.0); 1.49, 1.44 (d, H-11, 7.0) (Figure S17).

**^13^C-NMR** (75 MHz, DMSO-d6, δ ppm): 174.15, 169.27 (C-12); 160.56, 160.24 (C-15); 159.28, 157.96 (C-17); 148.08 (C-13); 140.56, 140.48 (C-8a); 140.01 (C-1a); 138.33, 138.26 (C-2); 131.12, 128.63 (C-19); 125.05, 124.96 (C-7); 123.59, 122.83 (C-14); 122.83 (C-5a); 122.77 (C-4a); 120.58, 120.18 (C-6); 120.42 (C-4); 119.68, 119.55 (C-5); 118.91, 118.76 (C-3); 112.32, 112.25 (C-8); 109.65, 109.60 (C-1); 107.85, 107.58 (C-18); 102.57, 102.34 (C-16); 44.26, 41.32 (C-10); 19.23, 18.89 (C-11) (Figure S18).

**FT-IR** (ATR in solid, ν cm^−1^): 3356m; 3224m; 3056w; 2980w; 1645vs; 1619vs; 1541sh; 1508s; 1445s; 1320m; 1227s; 1161m; 1116m; 1068m; 963m; 808m; 754m; 650w.

Chemical formula C_22_H_18_ClN_3_O_3_, exact mass 407.10367, **HRMS** (APCI+, DMSO+MeOH), *m*/*z* (%): 408.11047 (100) [M+H]^+^, 228.05774 (3) [C_14_H_11_ClN]^+^ (Figures S19 and S20).

**(*EZ*)-N’-(4-nitrobenzylidene)-2-(6-chloro-9*H*-carbazol-2-yl)propanehydrazide (1f)**; m.p. 235.6–238 °C; yield 74%.

The ratio between E and Z is 1.0:0.85.

**^1^H-NMR** (500 MHz, DMSO-d6, δ ppm, *J* Hz): 11.87, 11.61 (s, H-9); 11.39, 11.35 (s, HN); 8.26, 7.99 (s, H-13); 8.28, 8.26 (d, 2H, H-16, H-18, 9.8); 7.94, 7.91 (d, 2H, H-15, H-19, 9.8); 8.17, 8.13 (d, H-5, 1.9); 8.10, 8.06 (d, H-4, 8.2); 7.48 (bs, 1H, H-1); 7.47, 7.45 (d, 1H, H-8, 8.5); 7.35, 7.33 (dd, 1H, H-7, 1.9, 8.5); 7.20, 7.18 (d, H-3, 8.2); 4.83, 3.90 (q, H-10, 7.0); 1.50, 1.47 (d, H-11, 7.0) (Figure S21).

**^13^C-NMR** (125 MHz, DMSO-d6, δ ppm): 175.46, 170.31 (C-12); 147.75, 147.52 (C-17); 144.12, 140.19 (C-13); 140.67, 140.63 (C-8a); 140.55 (C-1a); 140.31, 139.80 (C-2); 138.33, 138.26 (C-14); 123.58 (C-5a); 122.85, 122.79 (C-4a); 120.47, 120.24 (C-6); 127.87, 127.60 (C-15, C-19); 124.02 (C-16, C-18); 125.10, 125.01 (C-7); 120.65, 120.56 (C-6); 120.47, 120.24 (C-4);119.70, 119.63 (C-5); 118.94, 118.75 (C-3); 112.35, 112.29 (C-8); 109.93, 109.66 (C-1); 44.52, 41.35 (C-10); 18.89, 18.53 (C-11) (Figure S22).

**FT-IR** (ATR in solid, ν cm^−1^): 3411w; 3313w; 3235w; 3048w; 2896w; 1655s; 1583m; 1555m; 1519vs; 1462m; 1342vs; 1273m; 1236m; 1192m; 1099w; 1068w; 950w; 844m; 737w.

Chemical formula C_22_H_17_ClN_4_O_3_, exact mass 420.09892, **HRMS** (APCI+, DMSO+MeOH), *m*/*z* (%): 421.10635 (70) [M+H]^+^, 391.13232 (6), 328.12149 (100) [C_18_H_19_ClN_3_O]^+^, 296.25864 (49), 282.27930 (15), 228.05769 (8) [C_14_H_11_ClN]^+^, 79.02095 (9) (Figures S23 and S24).

### 2.2. Tuberculostatic Activity

At the concentration of 4 mg/mL, the examined compounds demonstrated a greater inhibitory effect compared to 2 mg/mL. Among the tested compounds, **1d** and **1e** emerged as the most potent agents, effectively inhibiting the growth of all four strains of *M. tuberculosis*, whether susceptible or resistant, at 4 mg/mL. Particularly noteworthy was the efficacy of compounds **1b** and **1c**, which hindered the growth of three out of the four tested strains, including the *M. tuberculosis* strain resistant to INH. The lowest antimycobacterial activity was noted for the compounds **1a** and **1f** (Table 1).

## 3. Discussion

Owing to the escalating resistance of mycobacteria, there is a demand for innovative classes of antimycobacterial agents featuring novel mechanisms. Some findings in this domain could serve as promising foundations for subsequent studies aimed at developing new lead compounds to address multidrug-resistant tuberculosis.

To tackle this challenge, we integrated two pharmacophore fragments into a single molecule, comprising an NSAID-type carprofen structure and a hydrazone-type structure. This resulted in a novel series of carprofen-N-acyl hydrazone derivatives, which were then assessed for their tuberculostatic activity. The choice of the substituents on the N-acylhydrazonic fragment was influenced by the research carried out by Suyambulingam [63] and Gobis [64], who demonstrated the importance of substitution with electronegative groups (NO_2_, halogen, OH) for an increase in tuberculostatic activity. We found it interesting to study how these substituents with different electron-donating and electron-withdrawing effects can influence the antimycobacterial activity.

For the production of N-acyl hydrazones, we employed a microwave-assisted synthesis method. This approach offers advantages such as reduced reaction time, increased reaction yield, and enhanced purity of the final products by minimizing undesired side reactions when compared to conventional heating methods.

The compounds (**1a**–**f**) contain two geometrical stereoisomers, *E* and *Z*; the isomers interconvert easily to one another, this phenomenon making the separation process difficult.

The analysis of NMR spectra confirmed the existence of multiple signals due to geometrical stereoisomers *E* and *Z* or conformational synperiplanar and antiperiplanar stereoisomers found in the mixture. The presence of the chiral center at C-10 means that the number of diastereoisomers can be duplicated. For the N-acyl hydrazone group, -CO-NH-N=CH-, in ^1^H NMR spectra, -NH- gave a signal (singlet) at 11.38 ppm (for compound **1c**, as a unique signal) or presented two singlet signals for the other compounds, between 11.37 and 11.45 and 11.29 and 11.35 ppm for *syn* or *anti* stereoisomers. The proton of the =CH- moiety showed a singlet signal between 8.17 and 8.35 ppm and 7.87 and 8.21 ppm as an alternative between *syn* or *anti* stereoisomers for each compound. In carbon spectra, the signals of C12 and C13 exhibited doubling owing to the presence of *syn/anti*-conformational stereoisomerism. Thus, for the acyl hydrazone group, ^13^C NMR spectra showed two signals between 169.27 and 175.46 ppm due to C12 and between 132.90 and 148.02 ppm for C13. This phenomenon of duplication of signals caused by isomerism can be observed in Table S1.

**Figure 3:**
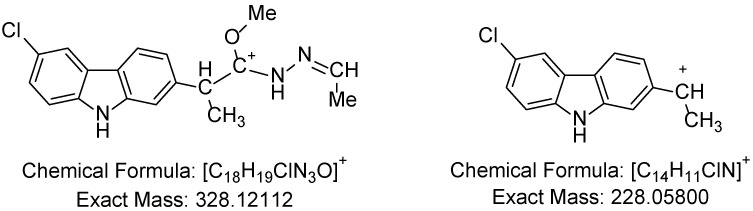
[C_18_H_19_ClN_3_O]^+^ and [C_14_H_11_ClN]^+^ cations.

The APCI+ high-resolution mass spectra were obtained using a mixture of DMSO+MeOH. The [M+H]^+^ molecular peaks were identified as base peaks for five substances, while the mass spectrum of **1f** was observed with a relative intensity of 70%. The presence of the molecular peaks as base peaks or with high relative intensity in the case of **1f** is evidence of the compounds’ identity and purity. The additional peaks identified in the mass spectra of the compounds align with the [C_18_H_19_ClN_3_O]+ and [C_14_H_11_ClN]+ cations at calculated *m*/*z* values of 328.12112 and 228.05800, for which the proposed structures are depicted on Figure 3. The two methyl groups attached in the fragment at a calculated *m*/*z* of 328.12112 probably result from the measurement in a methanolic solution.

The compounds were tested for their effectiveness against four microbial strains with varying susceptibility profiles with regard to RIF and INH.

The most active compounds were those bearing two fluorine or hydroxy substituents on the benzylidene fragment. Thus, **1b**, **1d**, and **1e** harbored two fluorine atoms (in positions 2, 6) or two hydroxy groups (in positions 2, 3 and 2, 4, respectively) on the benzylidene fragment. The compound **1c**, which also bears two fluorine atoms on the benzylidene fragment but in positions 3, 4, was less active than **1b**. This implies the significance of the positioning of identical substituents in relation to the rest of the molecule and to each other for the activity of these compounds, especially against the MDR strains. The lowest antimycobacterial activity was noted for the compounds **1a** and **1f**, bearing only one substituent on the benzylidene fragment situated in the *para* position.

## 4. Materials and Methods

### 4.1. Measurements

All reagents were obtained from Merck (Darmstadt, Germany) or Aldrich (Steinheim, Germany). The microwave-assisted synthesis was carried out using a Biotage^®^ Initiator Classic 2.0 system (Biotage, Uppsala, Sweden).

Melting points were determined using an Electrothermal 9100 apparatus (Bibby Scientific Ltd., Stone, UK) in open capillary tubes and were not corrected.

The IR spectra were analyzed with a Bruker Vertex 70 FT-IR spectrometer (Bruker Corporation, Billerica, MA, USA). Frequencies are expressed per cm^−1^ and were obtained using the ATR technique, and they are denoted as w (weak band), m (medium band), s (intense band), and vs (very intense band).

The ^1^H NMR and ^13^C NMR spectra were recorded in deuterated dimethyl sulfoxide (dmso-d6) using a Bruker Fourier 300 MHz instrument (Bruker Corporation, Billerica, MA, USA), operating at 300 MHz for ^1^H NMR and at 75 MHz for ^13^C NMR. Additionally, a Bruker Avance III 500 MHz instrument (Bruker Corporation, Billerica, MA, USA) was employed, operating at 500 MHz for proton and 125 MHz for carbon. In the NMR spectra, chemical shifts were recorded as δ values in parts per million (ppm) relative to tetramethylsilane as an internal standard. Coupling constants (J) are reported in hertz. Signal multiplicities are indicated as singlet (s), broad singlet (bs), doublet (d), broad doublet (bd), triplet (t), quartet (q), doublet of doublet (dd), and multiplet (m). The ^1^H NMR data are presented in the following order: chemical shifts, multiplicity, signal/atom attribution, and coupling constants. For ^13^C NMR data, the order is chemical shifts and then signal/atom attribution.

The APCI+ high-resolution mass spectra for compounds **1a**–**f** were recorded on a Thermo Scientific LTQ-Orbitrap XL spectrometer equipped with a standard ESI/APCI source. Thermo Xcalibur software (XcaliburTM 4.0.27.19 Software, Thermo Fisher Scientific, Waltham, MA, USA) was utilized for processing the mass spectra.

### 4.2. Chemistry

The syntheses of the compounds methyl (2*RS*)-2-(6-chloro-9*H*-carbazol-2-yl)-propanoate (carprofen methyl ester) (**3**) and (2*RS*)-2-(6-chloro-9*H*-carbazol-2-yl) propane hydrazide (carprofen hydrazide) (**4**) were presented in a previously published article [48].

For the synthesis of (*EZ*)-N’-benzylidene-2-(6-chloro-9*H*-carbazol-2-yl)propanehydrazide derivatives (**1a**–**f**), 2-(6-chloro-9*H*-carbazol-2-yl)propanehydrazide (**4**) (0.001 mol) and various aromatic aldehydes (0.001 mol) in 3 mL of absolute methanol and four drops of the catalyst (glacial acetyl acid) were introduced into a microwave tube. The reaction mixture underwent pre-stirring for 5 min, followed by irradiation for 25 min at 90 °C with very high absorption. Once the designated time elapsed, the mixture was cooled to room temperature and refrigerated overnight. The resulting product was then filtered and subjected to recrystallization from a 1:2 mixture of isopropanol and water.

### 4.3. Tuberculostatic Activity Assay

The tuberculostatic activity was evaluated against four strains of *M. tuberculosis*, including two susceptible to rifampicin (RIF) and isoniazid (INH) (encoded 2327 and 2337), one susceptible to RIF but resistant to INH (encoded 1762), and one resistant to both RIF and INH (encoded 309). The tested substances were incorporated into Lowenstein Jensen (LJ) solid medium at concentrations of 2 and 4 mg/mL. The tubes were then incubated at a 45° angle at 37 °C to ensure even distribution of the tested substances in the culture medium. After 48 h, the inoculum was seeded in the respective tubes, and incubation continued for 28 days.

For the sterility control of the culture medium and tested substances, LJ tubes supplemented with compounds **1a**–**f** at concentrations of 2 mg/mL and 4 mg/mL were left unseeded in the thermostat at 37 °C and observed for 28 days to confirm the absence of growth or contamination. The inoculum was seeded in LJ tubes and they were used as positive controls.

The colonies that emerged on the media containing varying concentrations of the tested substance were counted after an incubation period of 28 days.

For RIF and INH, the results were interpreted using the absolute concentration method, wherein the development of <20 colonies indicated susceptibility and >20 colonies indicated resistance to the respective antibiotic [65].

The conventional antibiotics were used at standard testing concentrations; namely, 40 μg/mL for RIF and 0.2 μg/mL for INH.

## 5. Conclusions

Ongoing research is addressing the rising challenge of drug resistance in tuberculosis, aiming to identify innovative compounds and enhance treatment approaches. In a broader context, structures resembling NSAIDs, such as carprofen, and hydrazone-like structures have been identified as important pharmacophores for designing potential therapeutics against *M. tuberculosis*. Six new derivatives of (EZ)-N’-benzylidene-2-(6-chloro-9*H*-carbazol-2-yl)propanehydrazide have been synthesized and assessed for their tuberculostatic activity against four strains of *M. tuberculosis.* Notably, these compounds exhibited a more pronounced inhibitory effect at a concentration of 4 mg/mL.

The double substitution with hydroxyl on the aromatic nucleus has proven to be particularly advantageous for the antimicrobial effect. Notably, compounds **1d** and **1e** demonstrated activity against all tested strains of *M. tuberculosis*.

Comprehensive research is essential to unravel the intricate mechanisms of action of these molecules. Additionally, evaluating their biocompatibility and bioavailability properties is imperative before advancing them to more advanced testing stages.

## Figures and Tables

**Table 1:** The count of M. tuberculosis colonies per standard inoculum volume (0.2 mL) retrieved from the culture medium supplemented with varying concentrations of the tested substances.

Mycobacterial Strain	Tested Compound	2 mg/mL	4 mg/mL	RIF	INH
*M. tuberculosis* 2327	**1a**	30	30	<20	<20
	**1b**	30–100	30–100	<20	<20
	**1c**	30	30	<20	<20
	**1d**	30	30	<20	<20
	**1e**	30–100	30	<20	<20
	**1f**	30–100	30–100	<20	<20
*M. tuberculosis* 2337	**1a**	30–100	30	<20	<20
	**1b**	30–100	30	<20	<20
	**1c**	30	30	<20	<20
	**1d**	30	30	<20	<20
	**1e**	30–100	30	<20	<20
	**1f**	30–100	30–100	<20	<20
*M. tuberculosis* 1762	**1a**	30–100	30–100	<20	>20
	**1b**	30–100	30	<20	>20
	**1c**	30–100	30	<20	>20
	**1d**	30–100	30	<20	>20
	**1e**	30–100	30	<20	>20
	**1f**	>100	30–100	<20	>20
*M. tuberculosis* 309	**1a**	30–100	30–100	>20	>20
	**1b**	30–100	30	>20	>20
	**1c**	30–100	30–100	>20	>20
	**1d**	30–100	30	>20	>20
	**1e**	30–100	30	>20	>20
	**1f**	>100	30–100	>20	>20

## Data Availability

The data presented in this study are available on request from the corresponding author.

## Supplementary Materials

The following supporting information can be downloaded at: https://www.mdpi.com/article/10.3390/antibiotics13030212/s1. Figure S1: The ^1^H-NMR spectra of (*EZ*)-N’-(4-bromobenzylidene)-2-(6-chloro-9*H*-carbazol-2-yl)propanehydrazide (**1a**). Figure S2: The ^13^C-NMR spectra of (*EZ*)-N’-(4-bromobenzylidene)-2-(6-chloro-9*H*-carbazol-2-yl)propanehydrazide (**1a**). Figure S3: The APCI+ MS spectrum of **1a** in DMSO+MeOH. Figure S4: The experimental (upper) and calculated (lower) APCI+ MS spectra of **1a.** Figure S5: The ^1^H-NMR spectra of (*EZ*)-N’-(2,6-difluorobenzylidene)-2-(6-chloro-9*H*-carbazol-2-yl)propanehydrazide (**1b**). Figure S6: The ^13^C-NMR spectra of (*EZ*)-N’-(2,6-difluorobenzylidene)-2-(6-chloro-9*H*-carbazol-2-yl)propanehydrazide (**1b**). Figure S7: The APCI+ MS spectrum of **1b** in DMSO+MeOH. Figure S8: The experimental (upper) and calculated (lower) APCI+ MS spectra of **1b.** Figure S9: The ^1^H-NMR spectra of (*EZ*)-N’-(3,4-difluorobenzylidene)-2-(6-chloro-9*H*-carbazol-2-yl)propanehydrazide (**1c**). Figure S10: The ^13^C-NMR spectra of (*EZ*)-N’-(3,4-difluorobenzylidene)-2-(6-chloro-9*H*-carbazol-2-yl)propanehydrazide (**1c**). Figure S11: The APCI+ MS spectrum of **1c** in DMSO+MeOH. Figure S12: The experimental (upper) and calculated (lower) APCI+ MS spectra of **1c.** Figure S13: The ^1^H-NMR spectra of (*EZ*)-N’-(2,3-dihydroxybenzylidene)-2-(6-chloro-9*H*-carbazol-2-yl)propanehydrazide (**1d**). Figure S14: The ^13^C-NMR spectra of (*EZ*)-N’-(2,3-dihydroxybenzylidene)-2-(6-chloro-9*H*-carbazol-2-yl)propanehydrazide (**1d**). Figure S15: The APCI+ MS spectrum of **1d** in DMSO+MeOH. Figure S16: The experimental (upper) and calculated (lower) APCI+ MS spectra of **1d.** Figure S17: The ^1^H-NMR spectra of (*EZ*)-N’-(2,4-dihydroxybenzylidene)-2-(6-chloro-9*H*-carbazol-2-yl)propanehydrazide (**1e**). Figure S18: The ^13^C-NMR spectra of (*EZ*)-N’-(2,4-dihydroxybenzylidene)-2-(6-chloro-9*H*-carbazol-2-yl)propanehydrazide (**1e**). Figure S19: The APCI+ MS spectrum of **1e** in DMSO+MeOH. Figure S20: The experimental (upper) and calculated (lower) APCI+ MS spectra of **1e.** Figure S21: The ^1^H-NMR spectra of (*EZ*)-N’-(4-nitrobenzylidene)-2-(6-chloro-9*H*-carbazol-2-yl)propanehydrazide (**1f**). Figure S22: The ^13^C-NMR spectra of (*EZ*)-N’-(4-nitrobenzylidene)-2-(6-chloro-9*H*-carbazol-2-yl)propanehydrazide (**1f**). Figure S23: The APCI+ MS spectrum of **1f** in DMSO+MeOH. Figure S24: The experimental (upper) and calculated (lower) APCI+ MS spectra of **1f.** Table S1: ^13^C-RMN chemical shifts for compounds **1a**–**f**.
