# Supplementary material for: Synthesis and Characterization of New N-acyl Hydrazone Derivatives of Carprofen as Potential Tuberculostatic Agents
Source: Antibiotics (Basel). 2024 Feb 23;13(3):212. doi: 10.3390/antibiotics13030212 (PMC10967372; doi:10.3390/antibiotics13030212)
Supplement: Supplementary file 1 [file antibiotics-13-00212-s001.zip › antibiotics-2851211-supplementary.pdf]

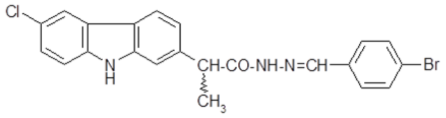

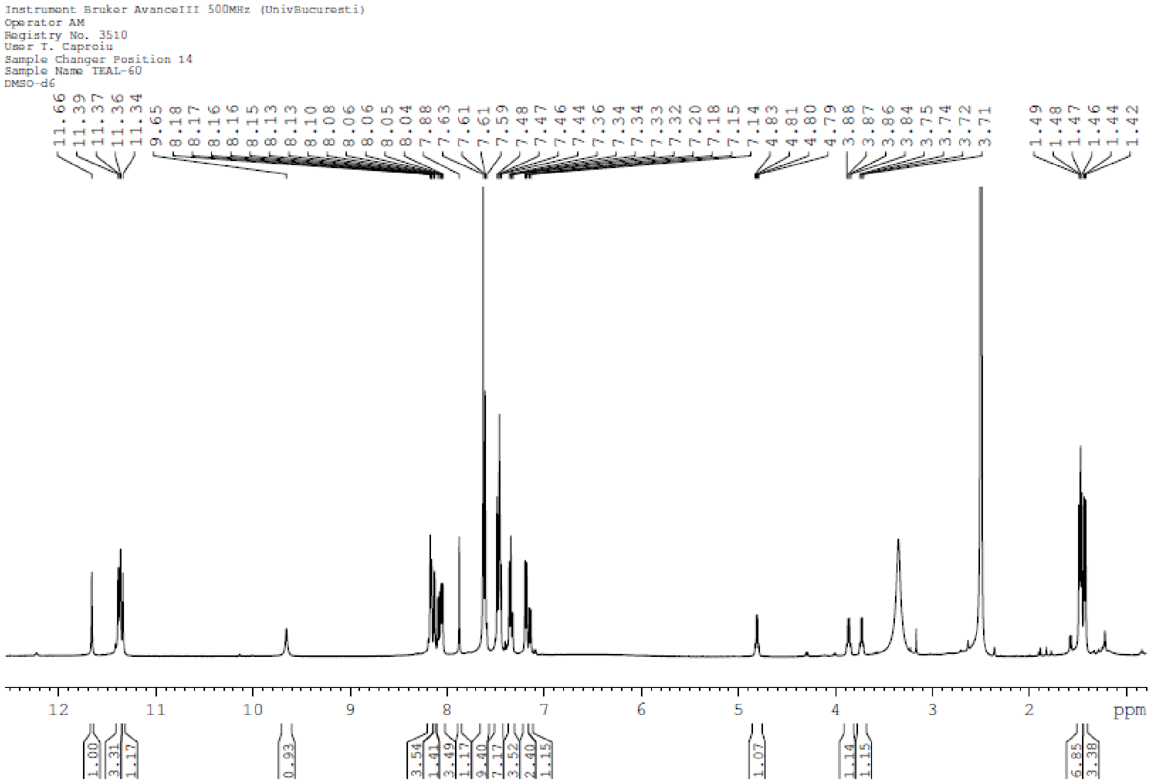

Figure S1: The ^{1}H-NMR spectra of **(*EZ*)-N'-(4-bromobenzylidene)-2-(6-chloro-9*H*-carbazol-2-yl)propanehydrazide (1a)**

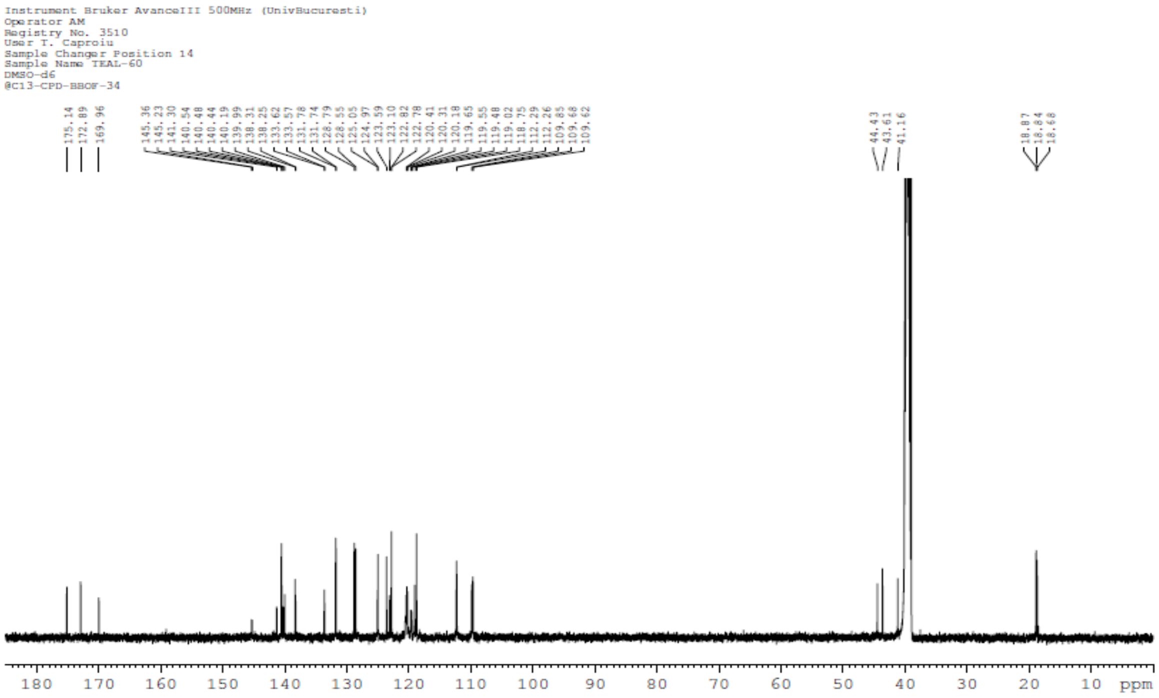

Figure S2: The ^{13}C-NMR spectra of **(*EZ*)-N'-(4-bromobenzylidene)-2-(6-chloro-9*H*-carbazol-2-yl)propanehydrazide (1a)**

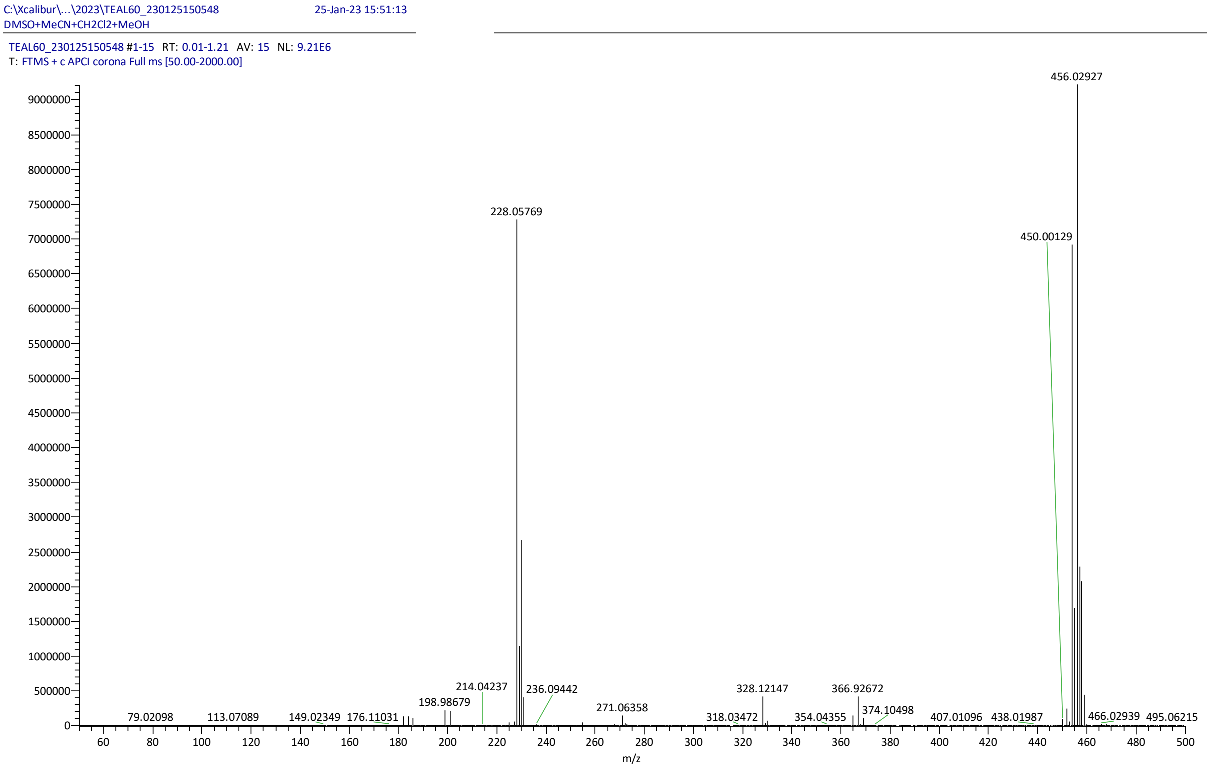

Figure S3:The APCI+ MS spectrum of **1a** in DMSO+MeOH

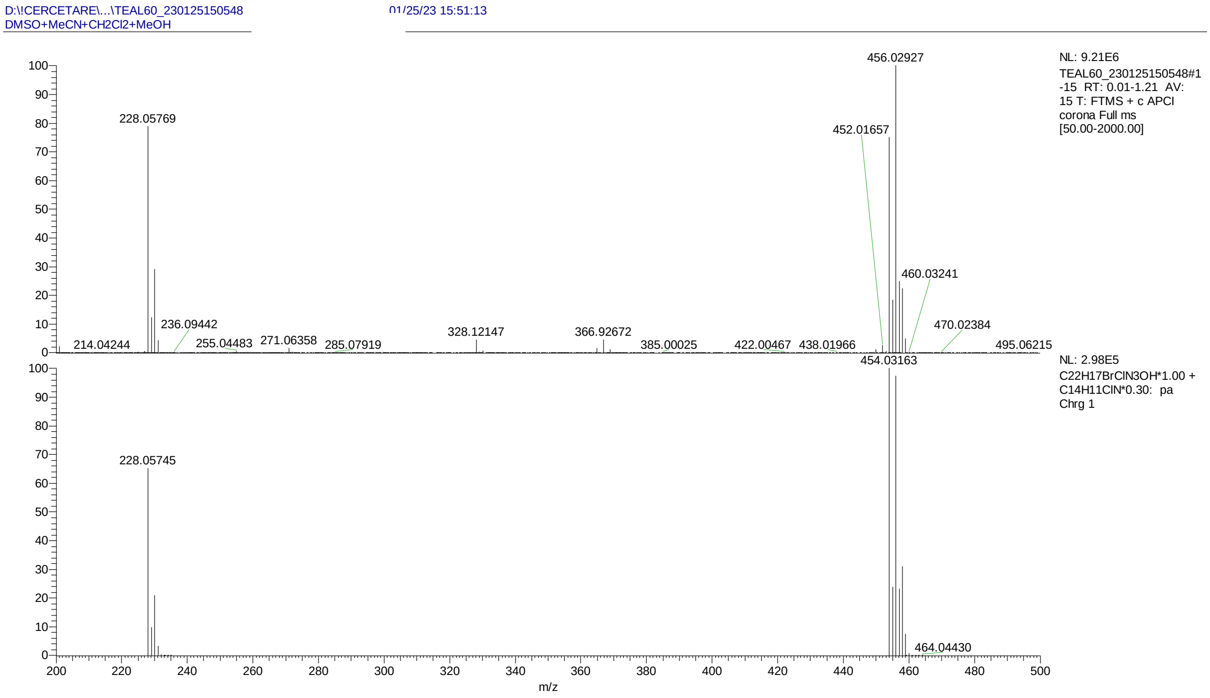

Figure S4:The experimental (up) andcalculated (down) APCI+ MS spectra of **1a**

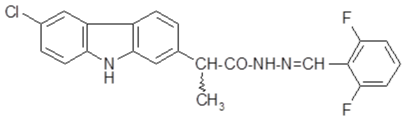

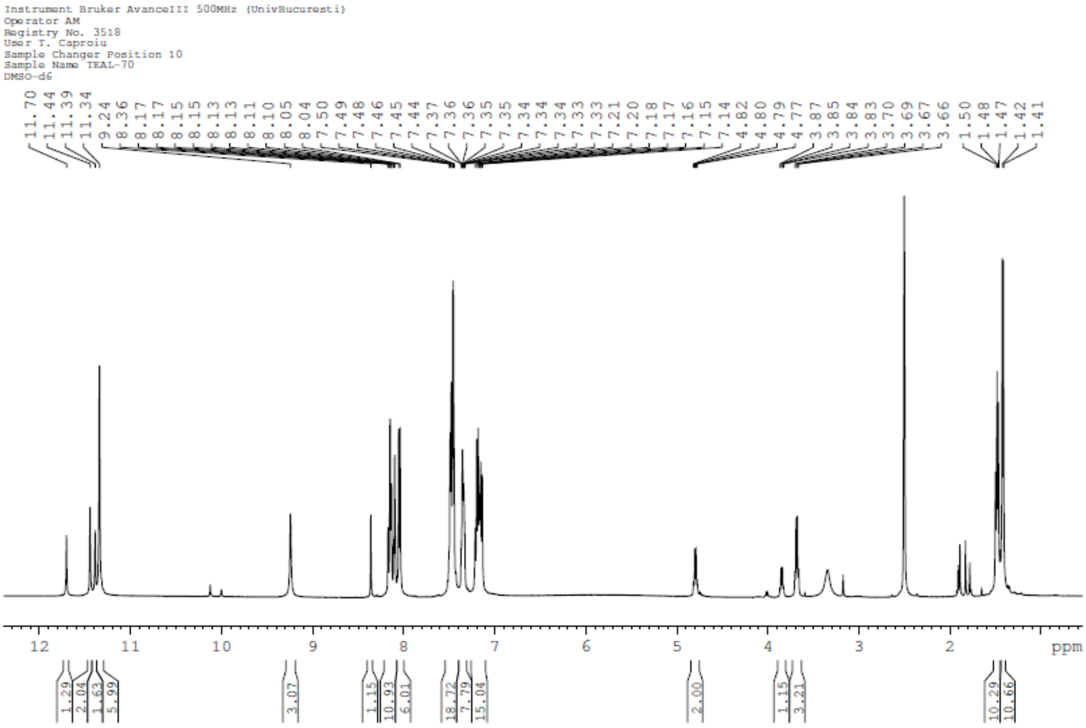

Figure S5: The ^{1}H-NMR spectra of **(*EZ*)-N'-(2,6-difluorobenzylidene)-2-(6-chloro-9*H*-carbazol-2-yl)propanehydrazide (1b)**

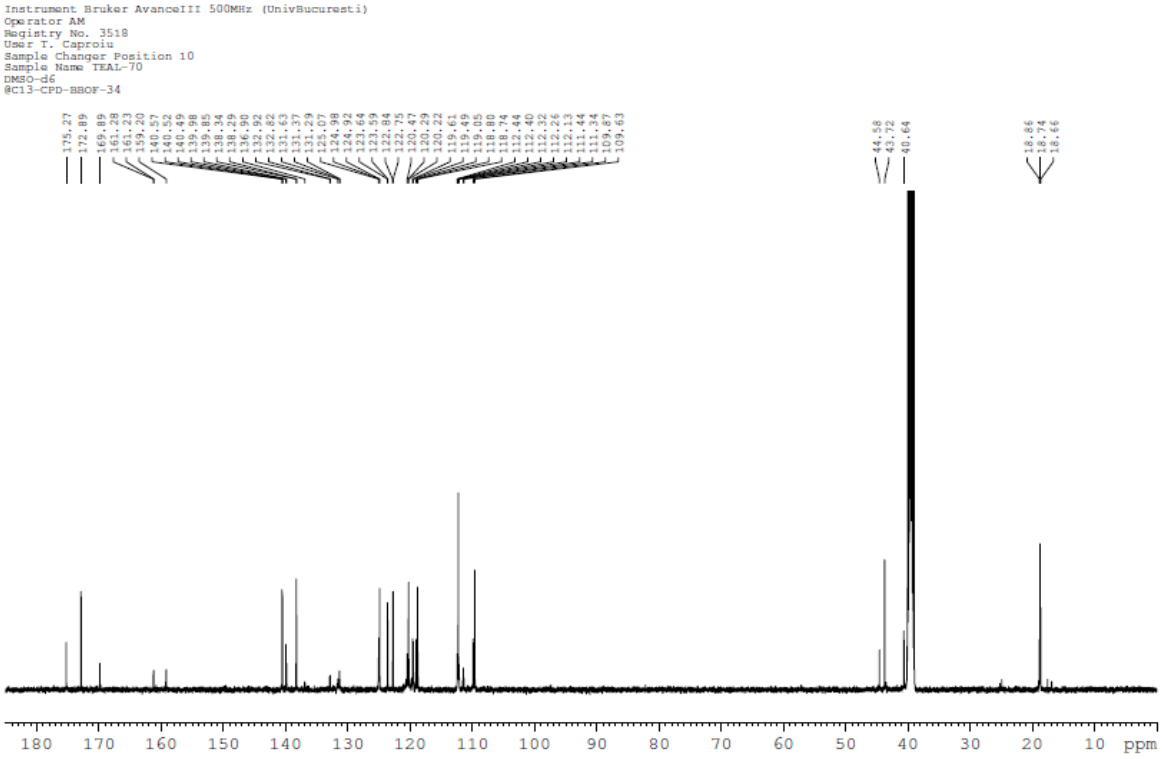

Figure S6: The ^{13}C-NMR spectra of **(*EZ*)-N'-(2,6-difluorobenzylidene)-2-(6-chloro-9*H*-carbazol-2-yl)propanehydrazide (1b)**

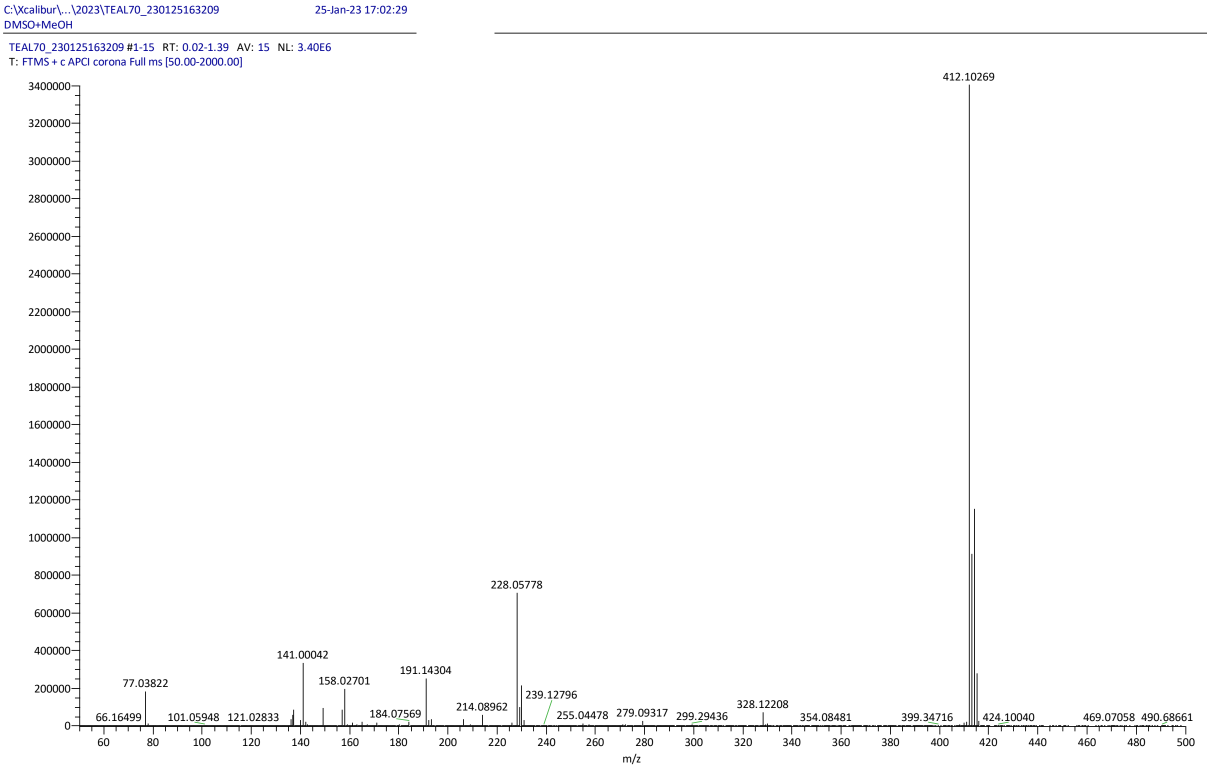

Figure S7:The APCI+ MS spectrum of **1b** in DMSO+MeOH

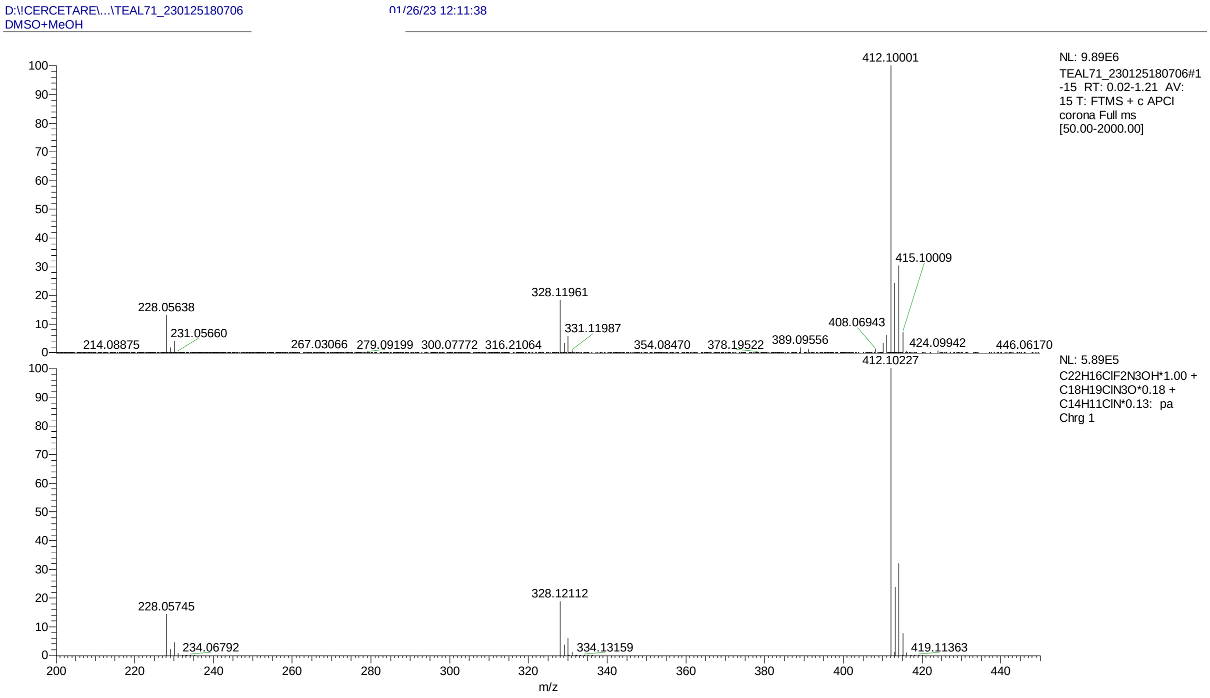

Figure S8:The experimental (up) andcalculated (down) APCI+ MS spectra of **1b**

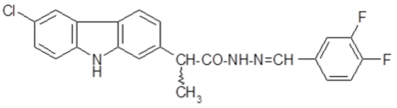

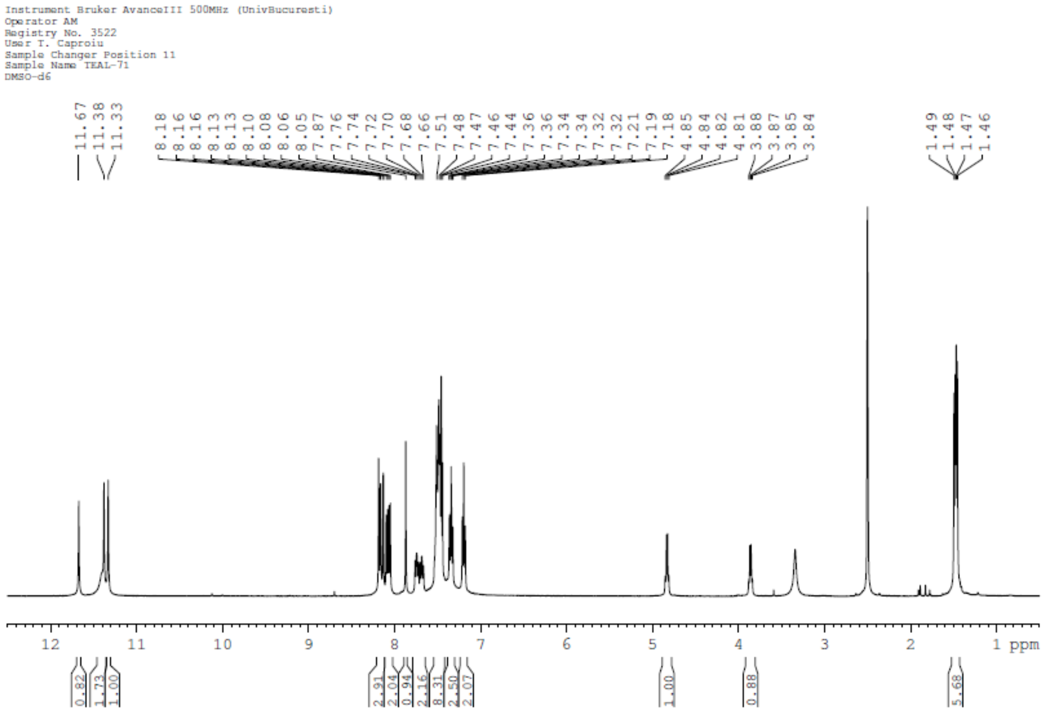

Figure S9: The ^{1}H-NMR spectra of **(*EZ*)-N'-(3,4-difluorobenzylidene)-2-(6-chloro-9*H*-carbazol-2-yl)propanehydrazide (1c)**

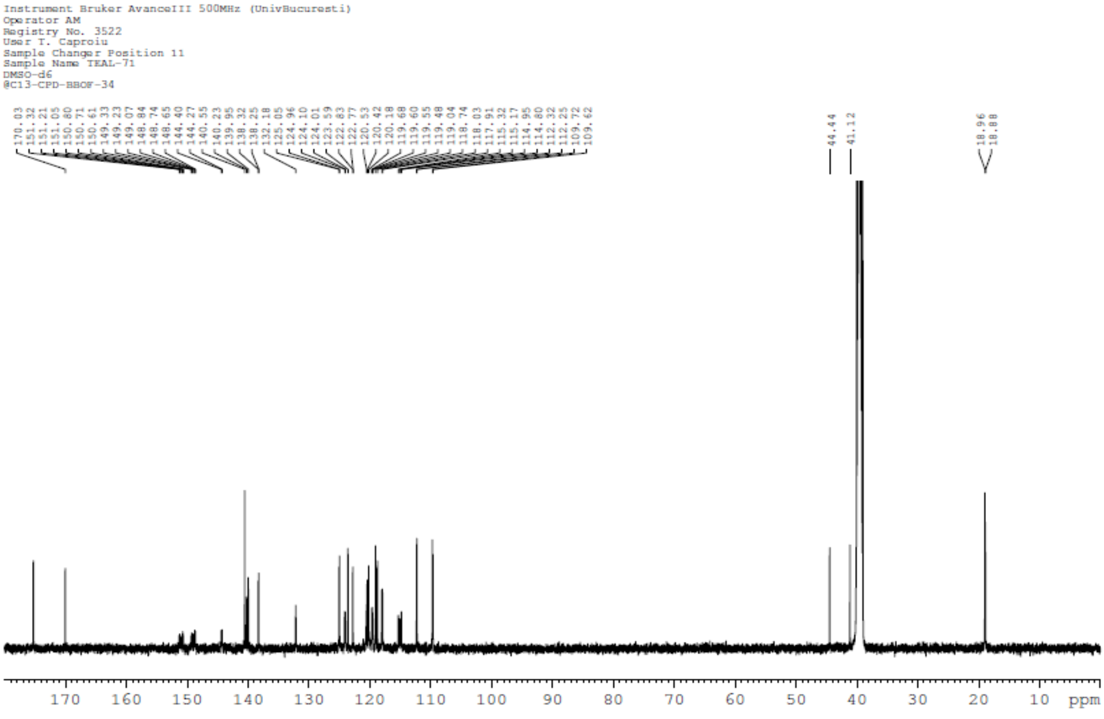

Figure S10: The ^{13}C-NMR spectra of **(*EZ*)-N'-(3,4-difluorobenzylidene)-2-(6-chloro-9*H*-carbazol-2-yl)propanehydrazide (1c)**

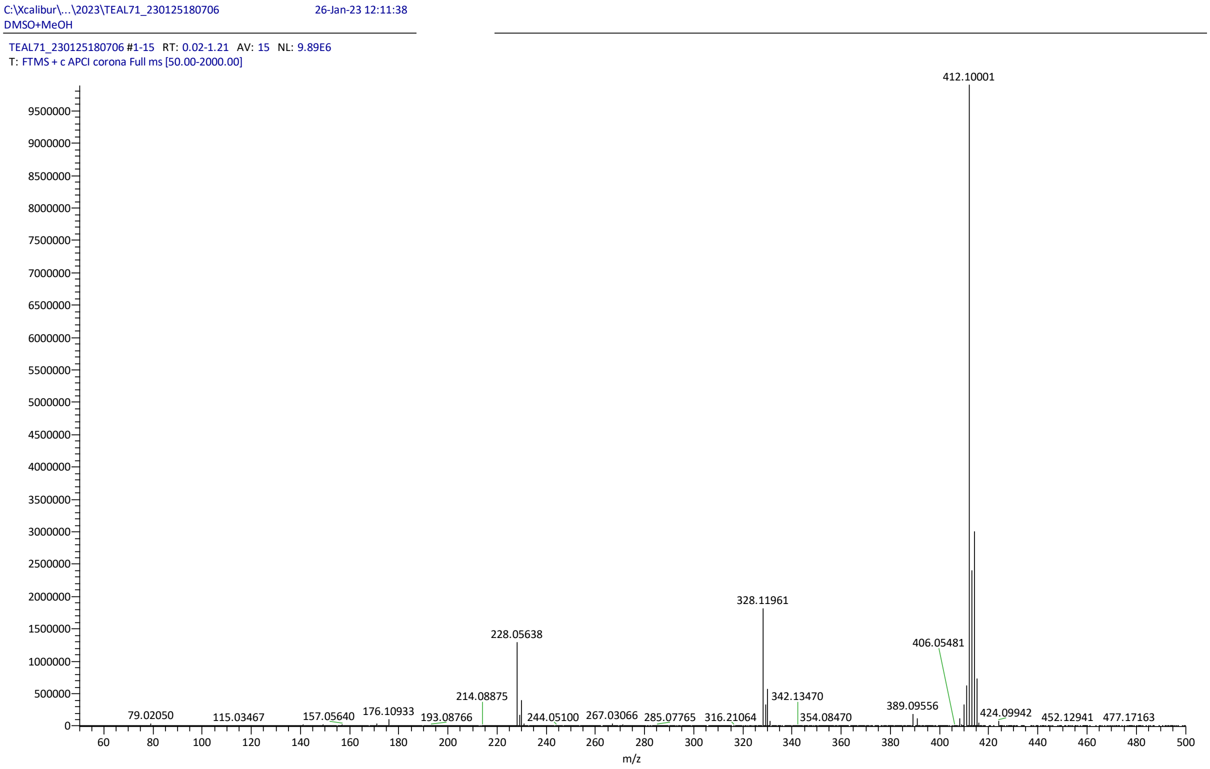

Figure S11:The APCI+ MS spectrum of **1c** in DMSO+MeOH

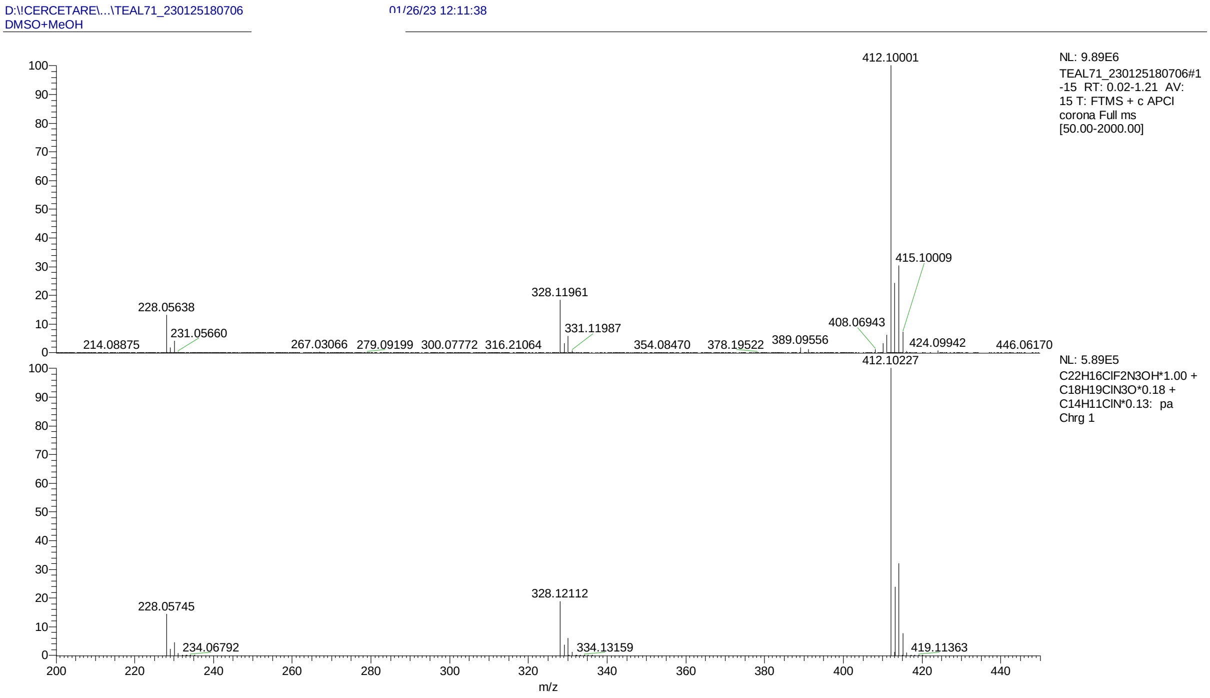

Figure S12:The experimental (up) andcalculated (down) APCI+ MS spectra of **1c**

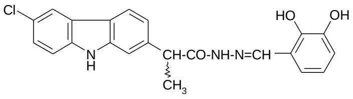

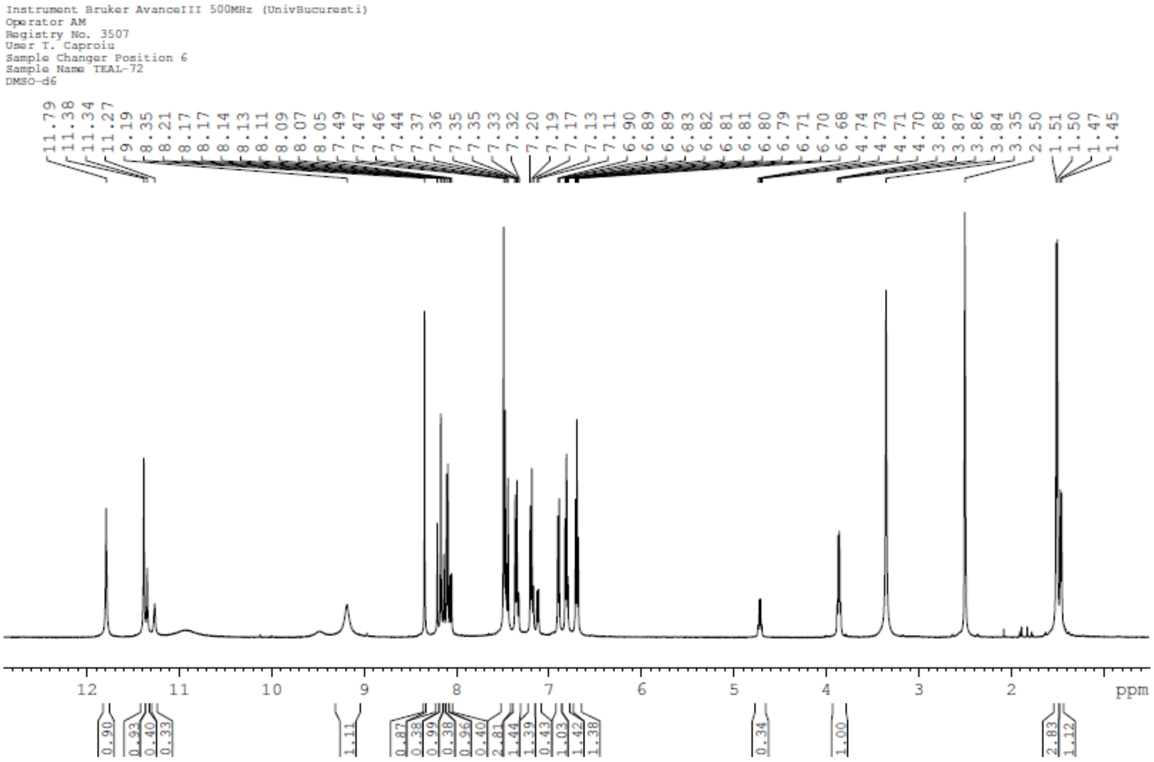

Figure S13: The ^{1}H-NMR spectra of **(*EZ*)-N'-(2,3-dihydroxybenzylidene)-2-(6-chloro-9*H*-carbazol-2-yl)propanehydrazide (1d)**

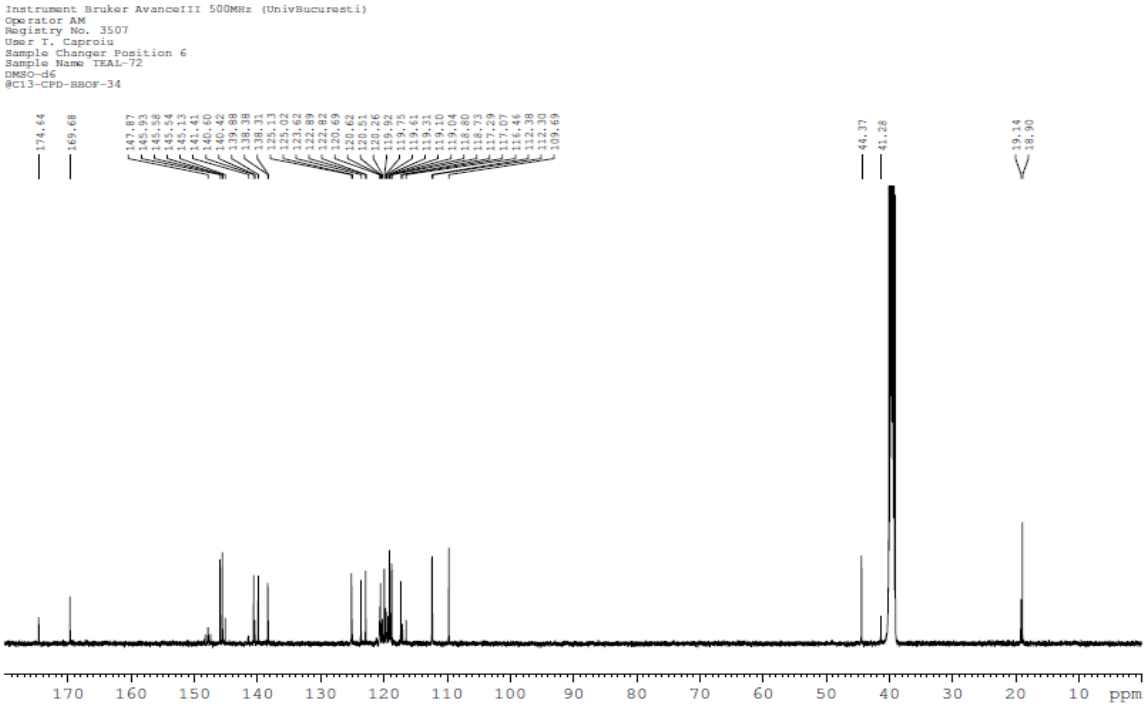

Figure S14: The ^{13}C-NMR spectra of **(*EZ*)-N'-(2,3-dihydroxybenzylidene)-2-(6-chloro-9*H*-carbazol-2-yl)propanehydrazide (1d)**

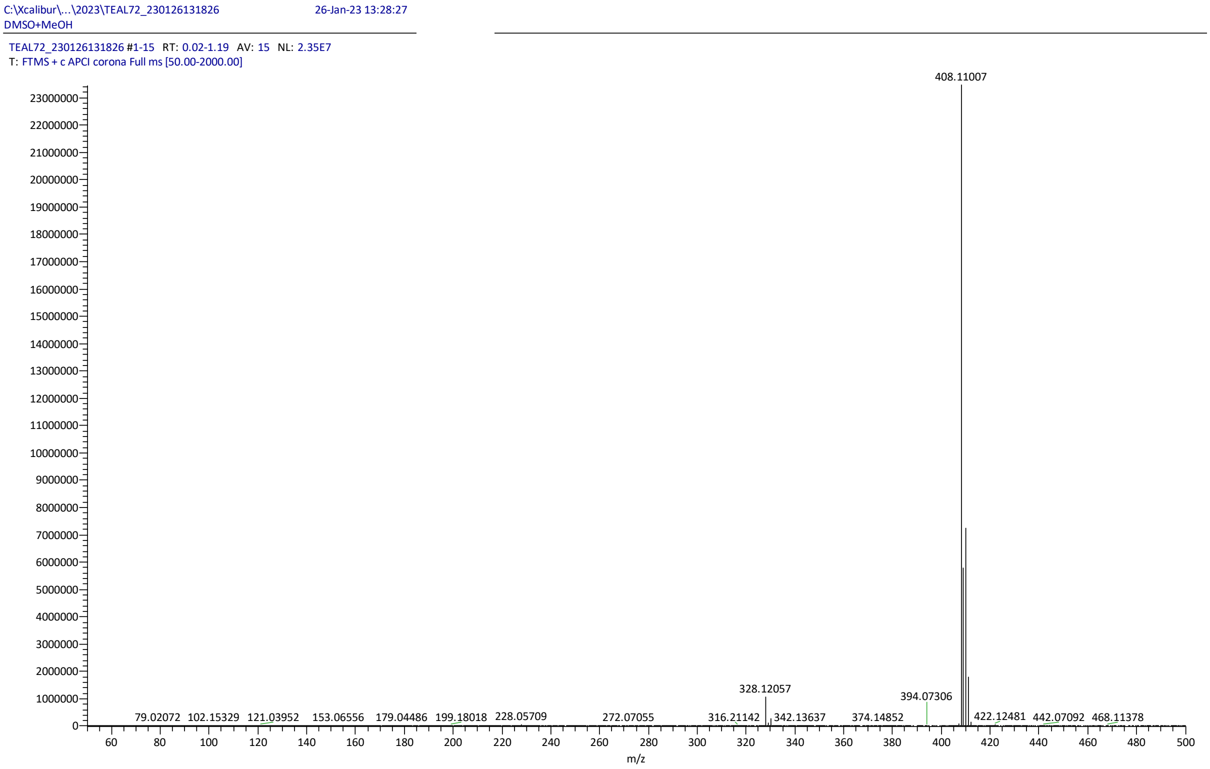

Figure S15:The APCI+ MS spectrum of **1d** in DMSO+MeOH

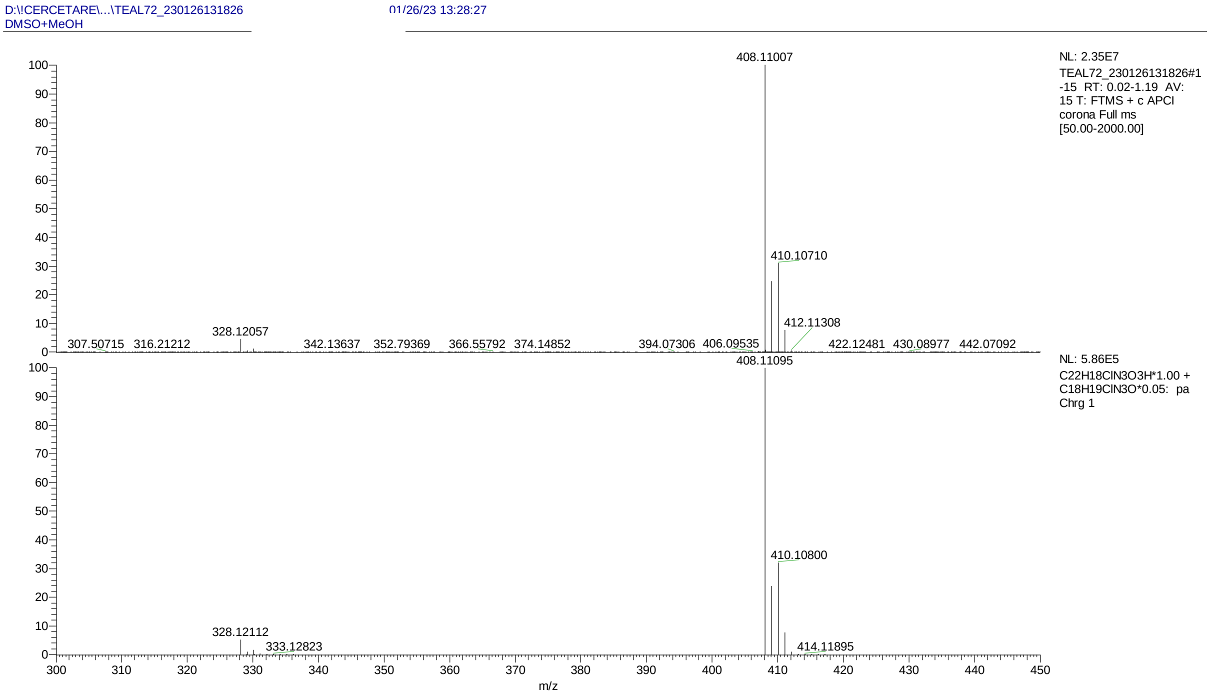

Figure S16:The experimental (up) andcalculated (down) APCI+ MS spectra of **1d**

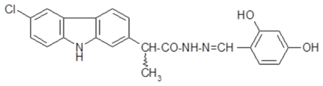

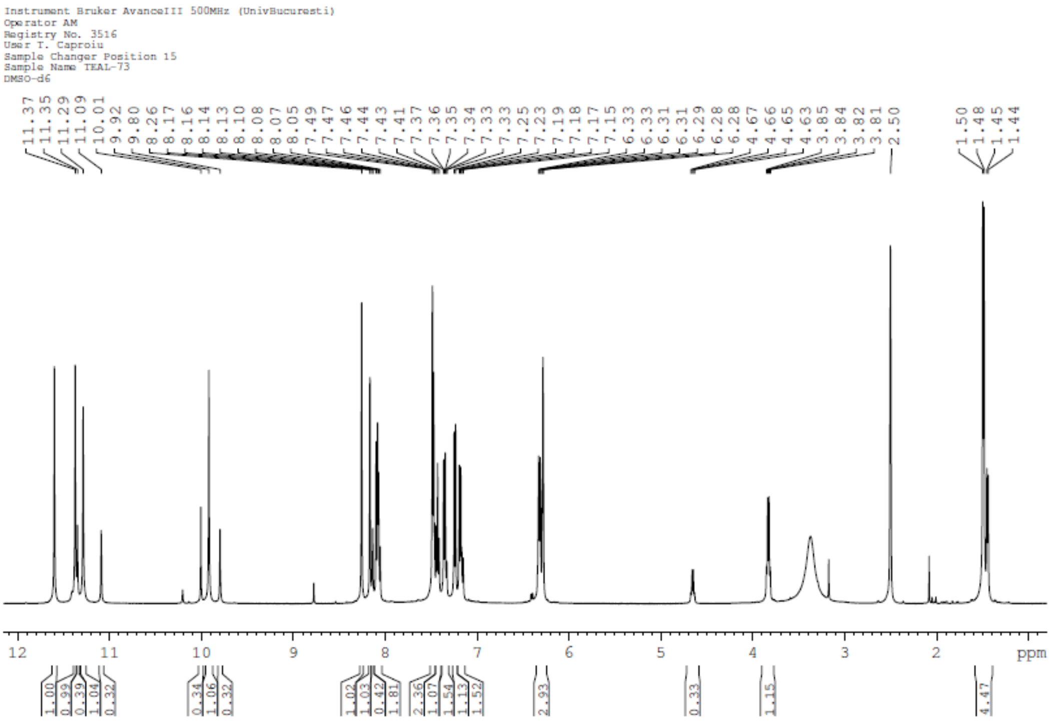

Figure S17: The ^{1}H-NMR spectra of **(*EZ*)-N'-(2,4-dihydroxybenzylidene)-2-(6-chloro-9*H*-carbazol-2-yl)propanehydrazide (1e)**

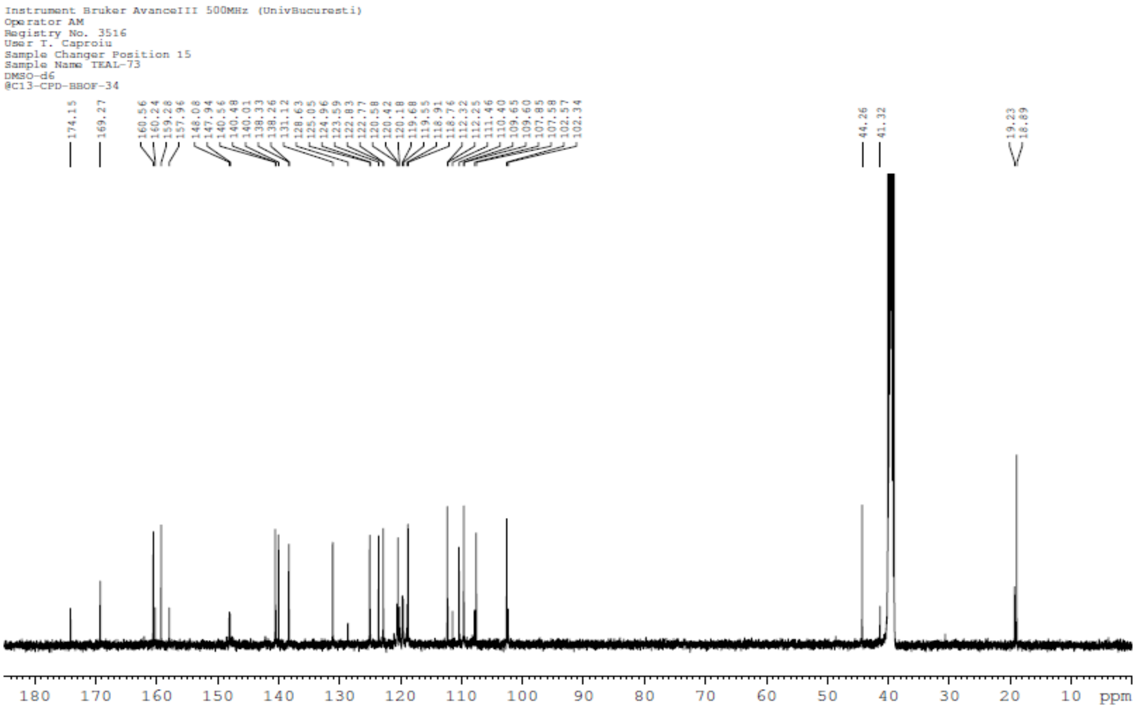

Figure S18: The ^{13}C-NMR spectra of **(*EZ*)-N'-(2,4-dihydroxybenzylidene)-2-(6-chloro-9*H*-carbazol-2-yl)propanehydrazide (1e)**

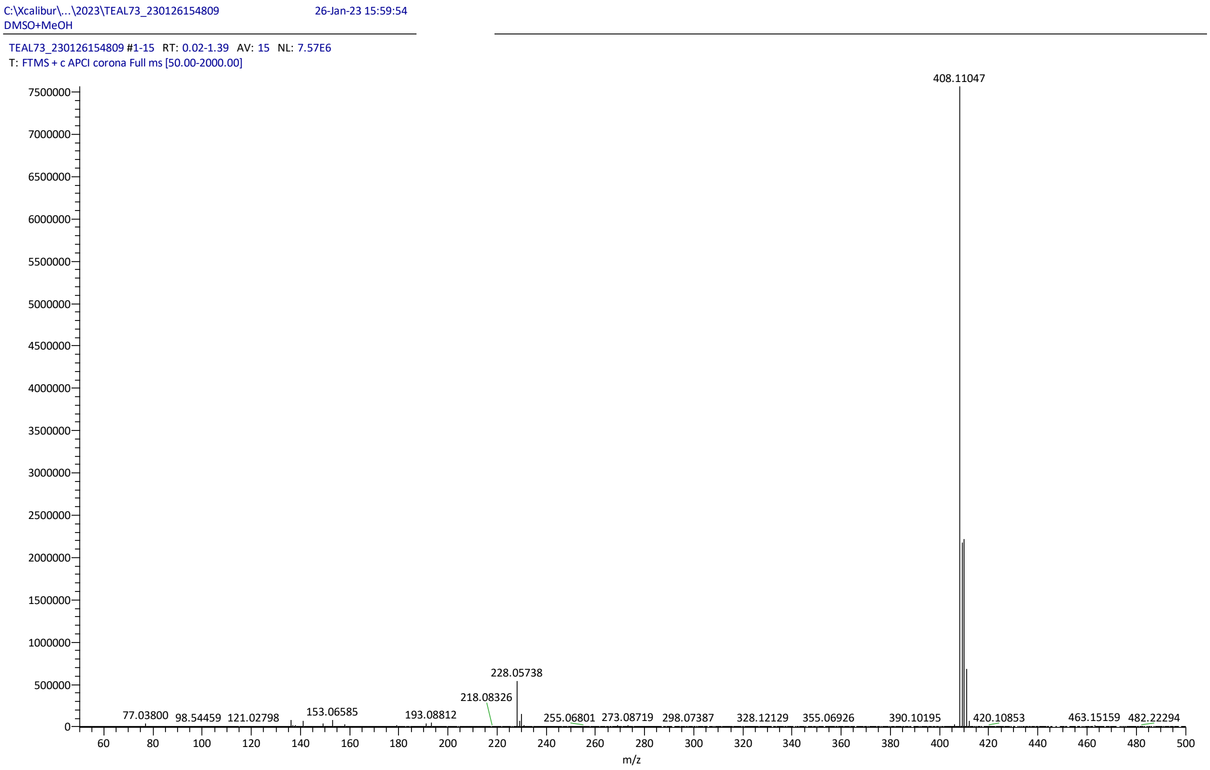

Figure S19: The APCI+ MS spectrum of **1e** in DMSO+MeOH

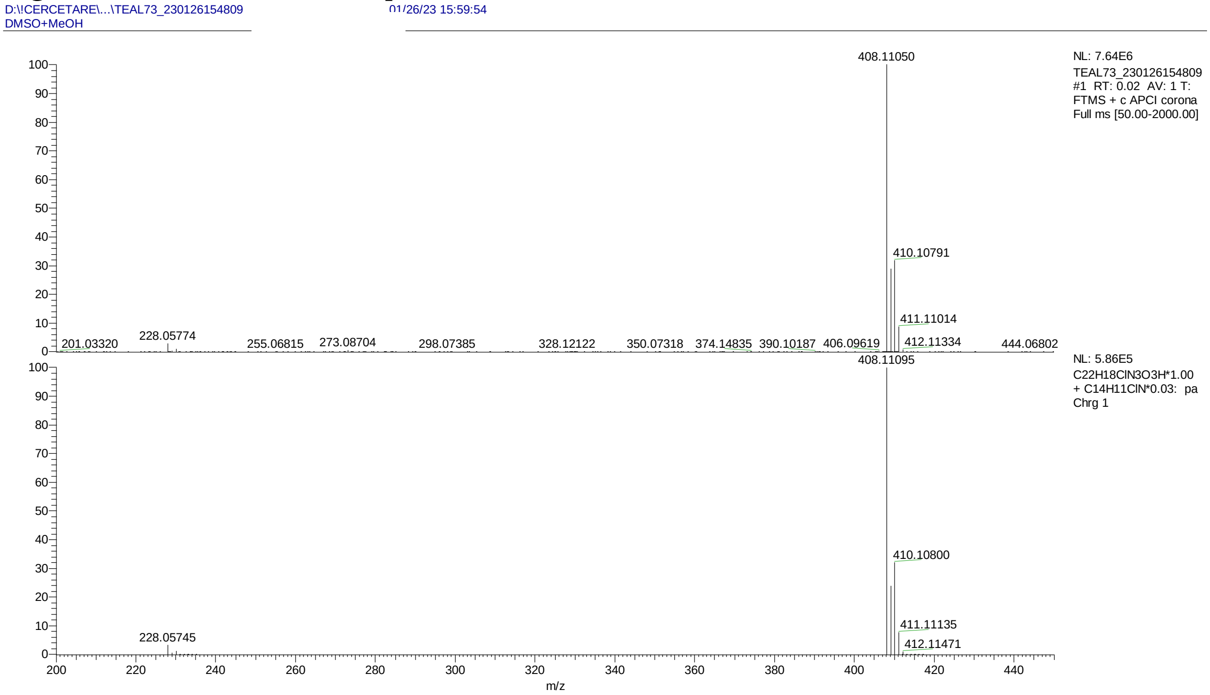

Figure S20: The experimental (up) andcalculated (down) APCI+ MS spectra of **1e**

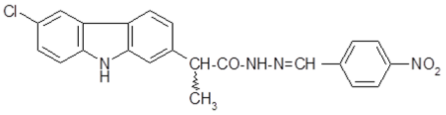

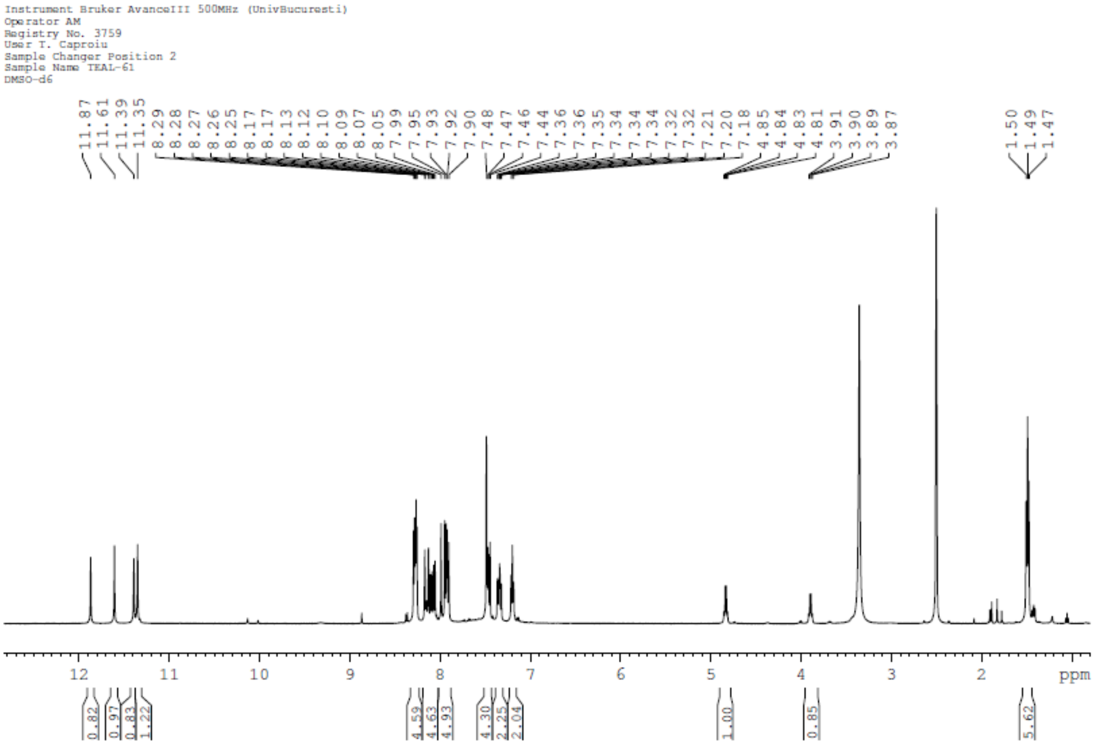

Figure S21: The ^{1}H-NMR spectra of ***EZ*)-N'-(4-nitrobenzylidene)-2-(6-chloro-9*H*-carbazol-2-yl)propanehydrazide (1f)**

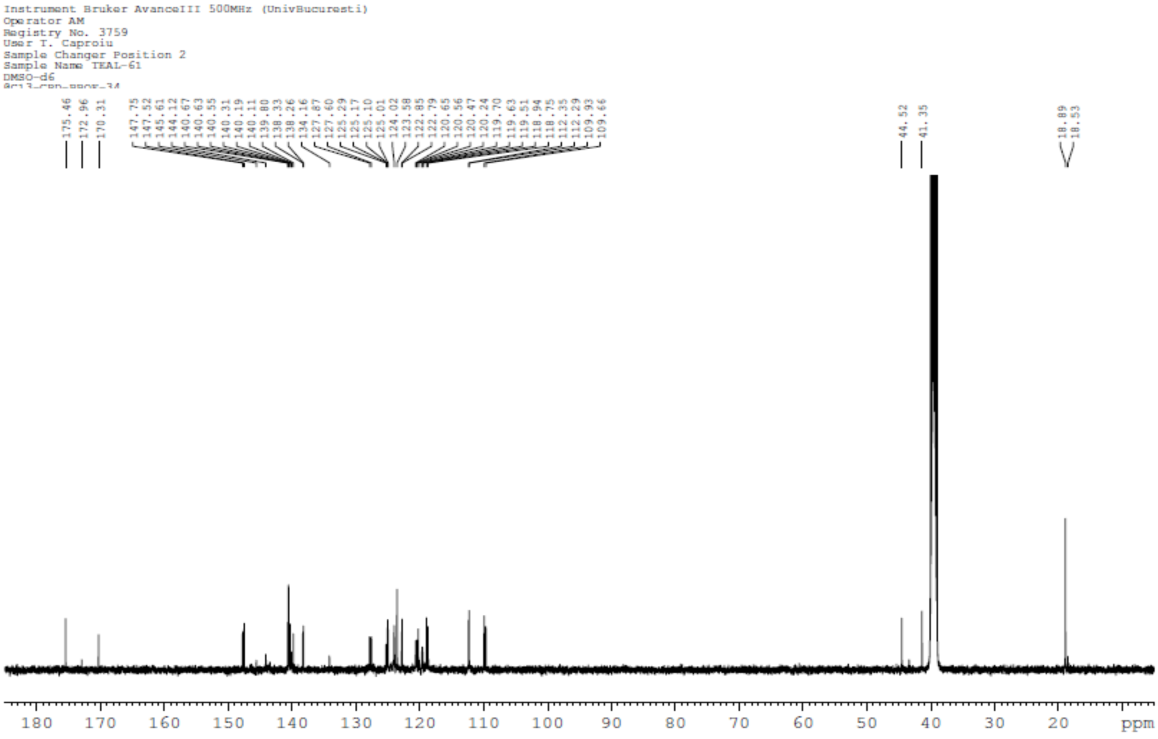

Figure S22: The ^{13}C-NMR spectra of ***EZ*)-N'-(4-nitrobenzylidene)-2-(6-chloro-9*H*-carbazol-2-yl)propanehydrazide (1f)**

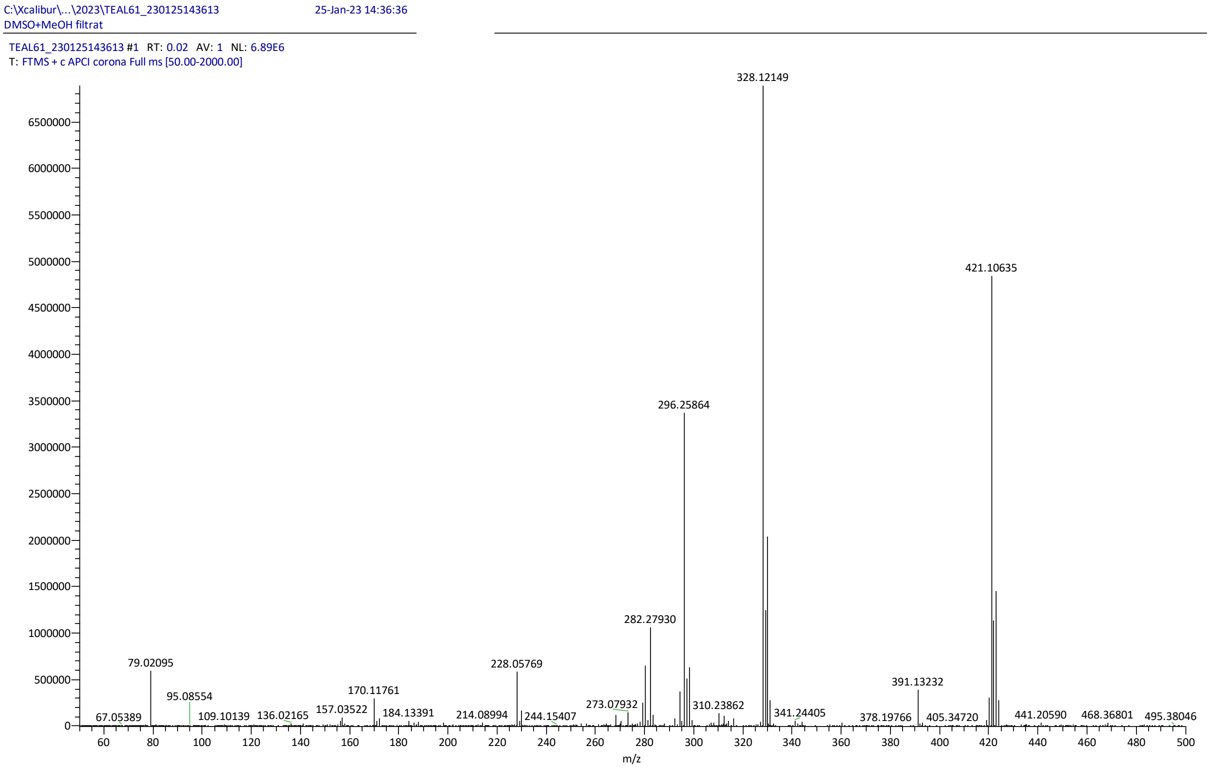

Figure S23: The APCI+ MS spectrum of **1f** in DMSO+MeOH

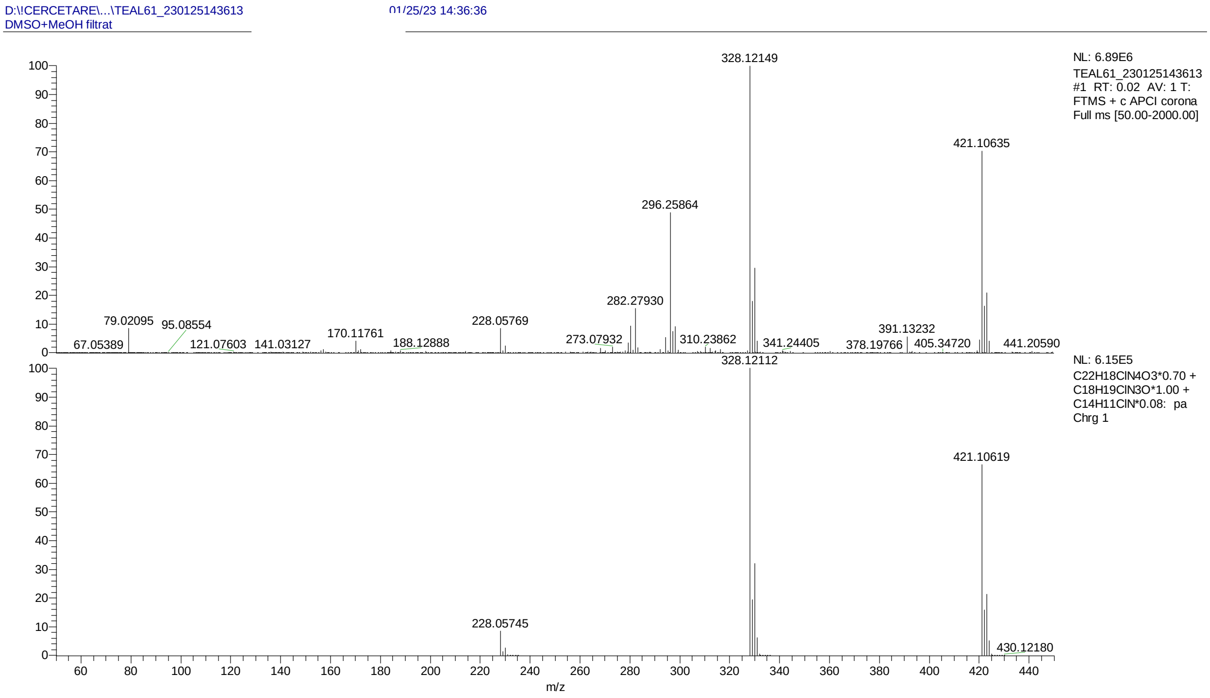

Figure S24: The experimental (up) andcalculated (down) APCI+ MS spectra of **1f**

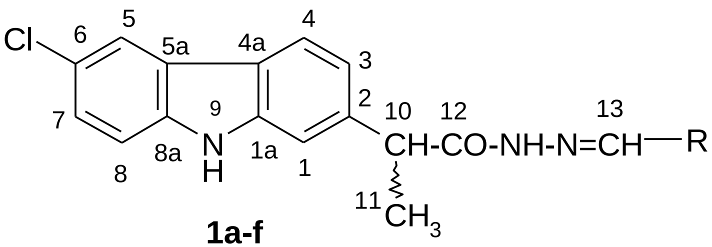

1a-f

Table S1. ^{13}C-RMN chemical shifts for compounds **1a-f** (T=303K; DMSO-d6)

Cmpd.	C - 1	C – 1a	C - 2	C - 3	C - 4	C – 4a	C - 5	C – 5a	C - 6	C - 7	C - 8	C – 8a	C – 10	C – 11	C- 12	C- 13
1a	109.85<br>109.62	140.44	138.31<br>138.25	119.02<br>118.75	119.68<br>120.18	122.82<br>122.78	119.55<br>119.48	123.59	120.41<br>120.31	125.05<br>124.97	112.29<br>112.26	140.54	44.43<br>43.61<br>41.26	18.84<br>18.68	175.14<br>172.89<br>169.96	145.36<br>141.30
1b	109.87<br>109.63	139.98<br>139.85	138.34<br>138.29	119.61<br>119.49	120.47	122.84<br>122.75	120.29<br>120.22	123.59	120.47<br>120.29	125.07<br>124.98	112.26	140.57<br>140.52	44.58<br>40.64	18.86<br>18.66	175.27<br>169.89	136.90<br>132.92
1c	109.72<br>109.62	139.95	138.32,<br>138.25	119.04<br>118.74	120.53	122.83<br>122.77	119.60<br>119.55	123.59	120.18<br>119.68	125.05<br>124.96	112.32<br>112.25	140.55	44.44<br>41.12	18.96<br>18.88	175.26<br>170.03	144.40<br>140.23
1d	109.69	139.88	138.38<br>138.31	118.99<br>118.73	119.75<br>119.31	122.89<br>122.82	119.92<br>119.61	123.62	120.51<br>120.26	125.13<br>125.02	112.38<br>112.30	140.60<br>140.42	44.37<br>41.28	19.14<br>18.90	174.64<br>169.68	147.87<br>141.41
1e	109.65<br>109.60	140.01	138.33<br>138.26	118.91<br>118.76	120.42	122.77	119.68<br>119.55	122.83	120.58<br>120.18	125.05<br>124.96	112.32<br>112.25	140.56<br>140.48	44.26<br>41.32	19.23<br>18.89	174.15<br>169.27	148.08
1f	109.93<br>109.66	140.55	140.31<br>139.80	118.94<br>118.75	120.47<br>120.24	122.85<br>122.79	119.70<br>119.63	123.58	120.65<br>120.56	125.10<br>125.01	112.35<br>112.29	140.67<br>140.63	44.52<br>41.35	18.89<br>18.53	175.46<br>170.31	144.12<br>140.19
